# Antibody CDR loops as ensembles in solution vs. canonical clusters from X-ray structures

**DOI:** 10.1080/19420862.2020.1744328

**Published:** 2020-04-07

**Authors:** Monica L. Fernández-Quintero, Martin C. Heiss, Nancy D. Pomarici, Barbara A. Math, Klaus R. Liedl

**Affiliations:** Institute of General, Inorganic and Theoretical Chemistry, and Center for Molecular Biosciences Innsbruck (CMBI), University of Innsbruck, Innsbruck, Austria

**Keywords:** Antibody design, canonical clusters, molecular dynamics simulations, conformational transitions, ensembles in solution

## Abstract

In the past decade, the relevance of antibodies as therapeutics has increased substantially. Therefore, structural and functional characterization, in particular of the complementarity-determining regions (CDRs), is crucial to the design and engineering of antibodies with unique binding properties. Various studies have focused on classifying the CDR loops into a small set of main-chain conformations to facilitate antibody design by assuming that certain sequences can only adopt a limited number of conformations. Here, we present a kinetic classification of CDR loop structures as ensembles in solution. Using molecular dynamics simulations in combination with strong experimental structural information, we observe conformational transitions between canonical clusters and additional dominant solution structures in the micro-to-millisecond timescale for all CDR loops, independent of length and sequence composition. Besides identifying all relevant conformations in solution, our results revealed that various canonical cluster medians actually belong to the same kinetic minimum. Additionally, we reconstruct the kinetics and probabilities of the conformational transitions between canonical clusters, and thereby extend the model of static canonical structures to reveal a dynamic conformational ensemble in solution as a new paradigm in the field of antibody structure design.

**Abbreviations:** CDR: Complementary-determining region; Fv: Antibody variable fragment; PCCA: Perron cluster analysis; tICA: Time-lagged independent component analysis; V_H_: Heavy chain variable region; V_L_: Light chain variable region

## Introduction

The importance of characterizing and engineering the structure of antibodies to improve specificity, stability and suitability as biotherapeutics has increased substantially in the past decades.^1–4^ Natural occurring antibodies are symmetric Y-shaped proteins, and each symmetric unit consists of a heavy and light chain. Sequence and structural diversity of antibodies is concentrated on six hypervariable loops, also known as complementarity-determining regions (CDRs), located within each of the two antibody antigen-binding domains. Three hypervariable loops (CDR-H1, CDR-H2, CDR-H3 and CDR-L1, CDR-L2, CDR-L3) are located on the heavy and light chain, respectively.^5^ Various studies have focused on classifying five of the six CDR loops into canonical conformations, except the CDR-H3 loop, assuming that, depending on the length and sequence composition, antibody CDR loops only adopt a limited number of main-chain conformations.^6^

The highest variability in length, sequence, and structure can be observed for the CDR-H3 loop. It is known that the CDR-H3 loop samples a large number of conformations during V(D)J recombination and somatic hyper-mutations.^7,8^ Together with the CDR-H3 loop, the CDR-L3 loop is situated in the center of the paratope and contributes to antigen recognition. The CDR-L3 loop reveals a comparable diversity to the CDR-H3 loop, but, without the contribution of a D gene, the degree of variability is lower.^9^ Besides the CDR-H3 and CDR-L3 loops, the CDR-H1 and CDR-H2 loops also play a vital role in many antibody–antigen interactions and are therefore targeted for mutagenesis in synthetic libraries. With the substantial rise in the number of antibody crystal structures, the number of antibody databases and sequence-based classification servers has also increased significantly.^10–13^ Numerous studies have tried to classify antibody CDR loops structurally and sequentially, and correlate them with their locus and sequence to improve fast antibody structure prediction and design.^13–16^ Additionally, several numbering systems for antibodies have been developed that are similar in the framework region but differ around the CDRs.^17–20^ The PyIgClassify database assigns conformational clusters by determining the CDR sequences and lengths using the international ImMunoGeneTics information system® nomenclature^18^ and calculating the dihedral angles φ and ψ of the residues in each CDR.^13^ Recent studies using molecular dynamics simulations extended the model of static canonical clusters to a characterization of the CDR-L3 and CDR-H3 loop as ensembles in solution by capturing several conformational transitions between different canonical structure medians.^21,22^

We analyzed the conformational diversity of all CDR loops to identify transition probabilities and timescales between canonical CDR loop conformations of the same length, and to kinetically characterize the CDR loop ensembles in the solution for all CDR loops. Therefore, we chose the median crystal structures of the high-populated canonical clusters with a good resolution and used this median X-ray structure as the starting point for molecular dynamics simulations.

## Results

### CDR-L1 loop ensemble in solution

**Figure 1.**
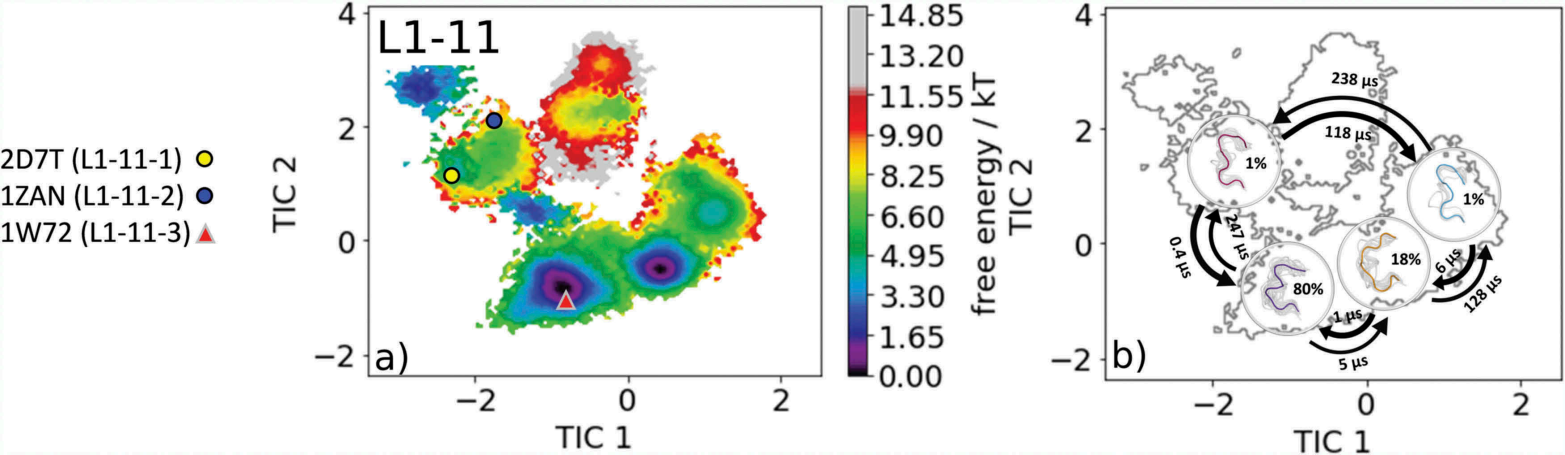
(a) Free energy surface of the CDR-L1 loop with a loop length of 11 residues including the projected canonical cluster median representatives. The canonical cluster representative used as starting structure for simulations is shaped as triangle, while all the other available canonical cluster median X-ray structures are visualized as circles and the respective color-coding is shown on the left. (b) Contours of the free energy surface are displayed in the background of the Markov-state model. The macrostate representatives with the respective macrostate ensemble and transition kinetics are also included. The macrostate representatives were colored independent of the canonical cluster representatives in (a) and summarize the kinetically relevant conformations of the CDR-L1 loop in solution. We obtained four macrostates, in which all canonical cluster medians are present, and we are even able to suggest two additional dominant solution structures.

The most common and highest populated CDR-L1 loop length observed in antibody crystal structures is 11 residues. With this loop length, there exist three canonical clusters L1-11-1, L1-11-2 and L1-11-3. As the starting structure for our simulations, we chose the antigen-binding fragment (Fab) binding to the tumor-associated human leukocyte antigen (HLA) HLA-A1.MAGE-A1 (Protein Data Bank (PDB) accession code: 1W72), which is the median crystal structure of the canonical L1-11-3 cluster and is the only one of the three available canonical clusters composed of lambda light chains.^23^ As described in the methods section, we clustered the obtained 1 µs metadynamics simulation and used the resulting 132 cluster representatives as starting structures for each 100 ns simulations. This approach does not only allow the generation of a broad ensemble of CDR-L1 loop conformations but also the characterization of the ensemble in solution kinetically. Figure 1a shows the obtained free energy surface of 13.2 µs trajectories with the projection of the available canonical cluster medians (2D7 T, 1ZAN, 1W72) into the time-lagged independent component analysis (tICA) space. We observed that the 1W72 median crystal structure lies in the global free energy minimum in solution, while the two highest populated cluster medians of the L1-11-1 and L1-11-2 canonical clusters lie in the same local shallow side minimum. Besides sampling all available canonical CDR-L1 loop conformations, we observed other dominant minima in a solution that should be considered when characterizing the CDR-L1 loop ensemble. Figure 1b illustrates the macrostate representatives with the respective macrostate ensemble in the background and the broadness of the ensemble are in agreement with the calculated state probabilities. The transitions between the two macrostates, in which all available canonical structures are present, occur in the high microsecond timescale, while the transition between the dominant solution minima occurs in the nanosecond timescale. SI Figure S1 illustrates an overlay of the canonical cluster median crystal structures, which lie in the same kinetic minimum with the obtained CDR-L1 loop ensemble in solution.

### CDR-L2 loop ensemble in solution

**Figure 2.**
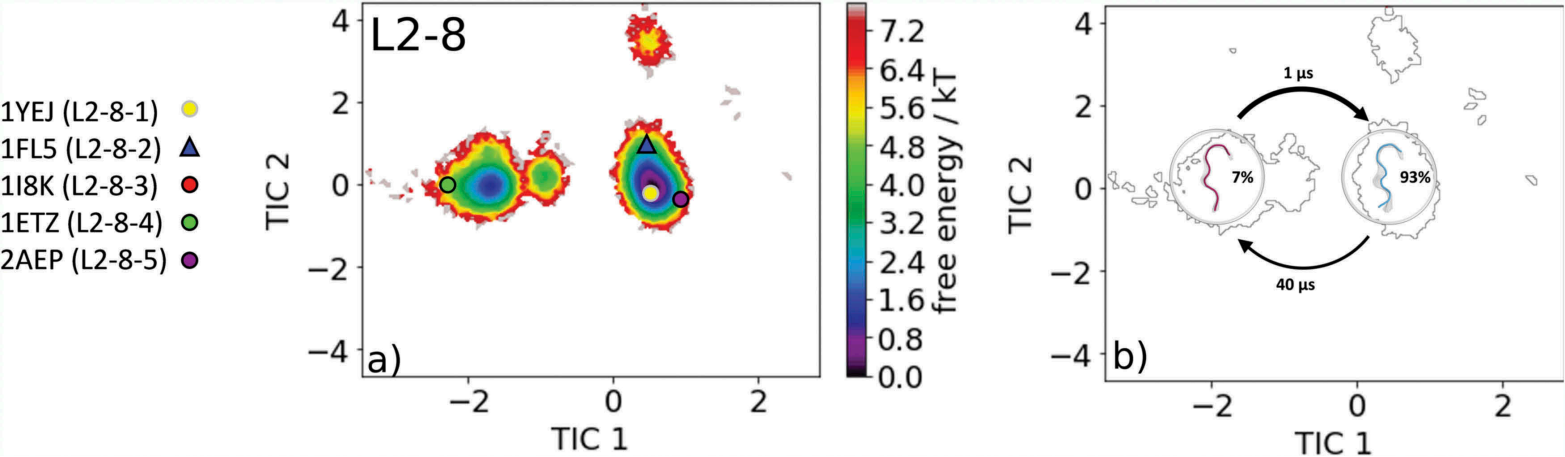
(a) Free energy surface of the CDR-L2 loop with a loop length of eight residues including the projected canonical cluster median representatives. The canonical cluster representative used as starting structure for simulations is shaped as triangle, while all the other available canonical cluster median X-ray structures are visualized as circles and the respective color-coding is shown on the left. (b) Contours of the free energy surface are displayed in the background of the Markov-state model. The macrostate representatives with the respective macrostate ensemble and transition kinetics are also included. The macrostate representatives were colored independent of the canonical cluster representatives in (a) and summarize the kinetically relevant conformations of the CDR-L2 loop in solution. We obtained two macrostates, in which all canonical cluster medians are present and the highest populated cluster representative directly lies in the global free energy minimum in solution.

**Figure 3.**
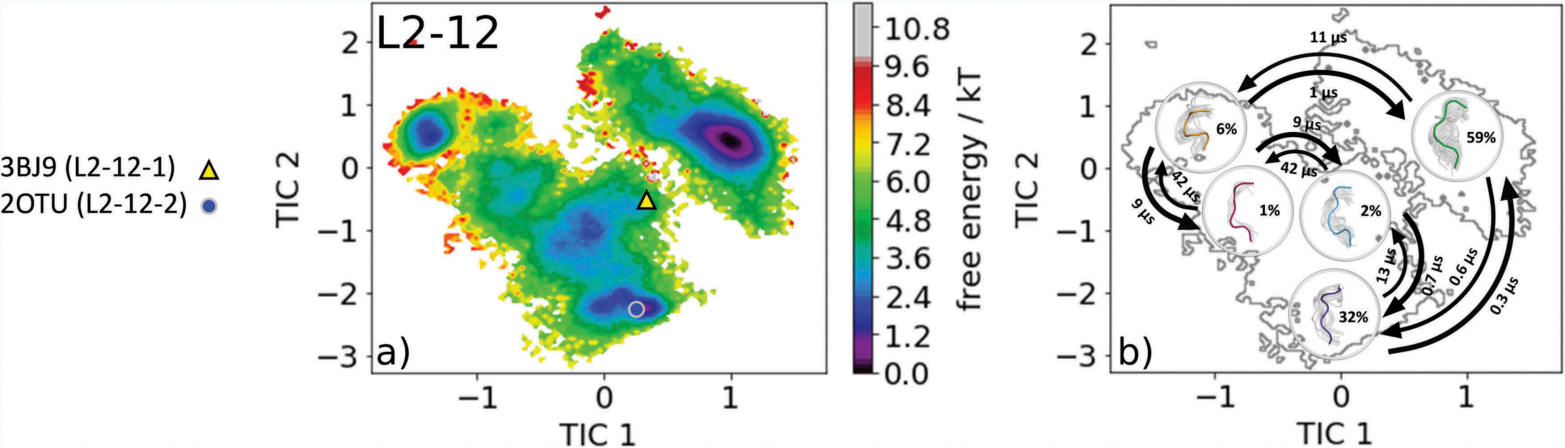
(a) Free energy surface of the CDR-L2 loop with a loop length of 12 residues including the projected canonical cluster median representatives. The canonical cluster representative used as the starting structure for simulations is shaped as triangle, while the other available canonical cluster median X-ray structure is visualized as circle and the respective color-coding is shown on the left. (b) Contours of the free energy surface are displayed in the background of the Markov-state model. The macrostate representatives with the respective macrostate ensemble and transition kinetics are also included. The macrostate representatives were colored independent of the canonical cluster representatives in (a) and summarize the kinetically relevant conformations of the CDR-L2 loop in solution. We obtained five macrostates, in which all canonical cluster medians are present.

The most common and highest populated CDR-L2 loop length found in the PDB is eight residues. With this specific loop length, there exist five different canonical clusters L2-8-1, L2-8-2, L2-8-3, L2-8-4, and L2-8-5. As the starting structure for our simulations, we chose the Fab of the catalytic 28B4 antibody, which is the median crystal structure of the L2-8-2 canonical cluster (PDB code: 1FL5). The 28B4 antibody catalyzes a periodate-dependent oxidation of sulfide to sulfoxide.^24^ Following the procedure described in the methods section, the obtained 126 cluster representatives of the metadynamics simulations were simulated for every 100 ns molecular dynamics simulations. To identify the kinetics and thermodynamics of the CDR-L2 loop ensemble in solution, we calculated a Markov-state model based on a tICA by using the backbone torsions of the CDR-L2 loop (Figure 2). We obtained a fully connected Markov-state model with two macrostates in which four of the five available canonical cluster structures with the loop length of eight residues were present. Figure 2a reveals that within our global minimum in solution we identified three canonical cluster median PDBs (1YEJ, 2AEP, and 1FL5) and the highest populated canonical cluster representative 1YEJ lies directly in our global free energy minimum. The lowest populated canonical cluster L2-8-3 representative (1I8 K) was not observed for this sequence. Figure 2b illustrates the macrostate representatives with the respective ensemble in the background and the transition timescales between different canonical structures and ensembles in solution. SI Figure S2 shows an overlay of the CDR-L2 loop ensemble in solution with the canonical cluster median structures belonging to the same kinetic minimum in solution. Besides the CDR-L2 loop length of eight residues, there are only two additional canonical clusters with the loop length of 12 residues L2-12-1 and L2-12-2. As the starting structure for our simulations, we chose the single-chain surrogate light chain variable domain, which is a key regulator of B cell development in the bone marrow and represents the median crystal structure of the L2-12-1 canonical cluster (PDB accession code: 3BJ9). Figure 3a illustrates the free energy surface of the CDR-L2 loop in the tICA space of 18.0 µs trajectories with the projected canonical cluster median crystal structures (PDB accession codes: 2OTU and 3BJ9).^25^ The results clearly show that besides sampling canonical cluster transitions, other dominant solution structures should be considered when characterizing the CDR-L2 loop ensemble in solution. The Markov-state model in Figure 3b reveals fast conformational transitions in the low µs timescale within the dominant conformations in solution, which strongly suggests that the dominant solution structure should be included when designing CDR-L2 loop with a loop length of 12 residues. Again, SI Figure S3 shows an overlay of the available canonical cluster median structures with the obtained CDR-L2 loop ensemble in solution combined with the contours of the free energy landscape in the background.

### CDR-L3 loop ensemble in solution

**Figure 4.**
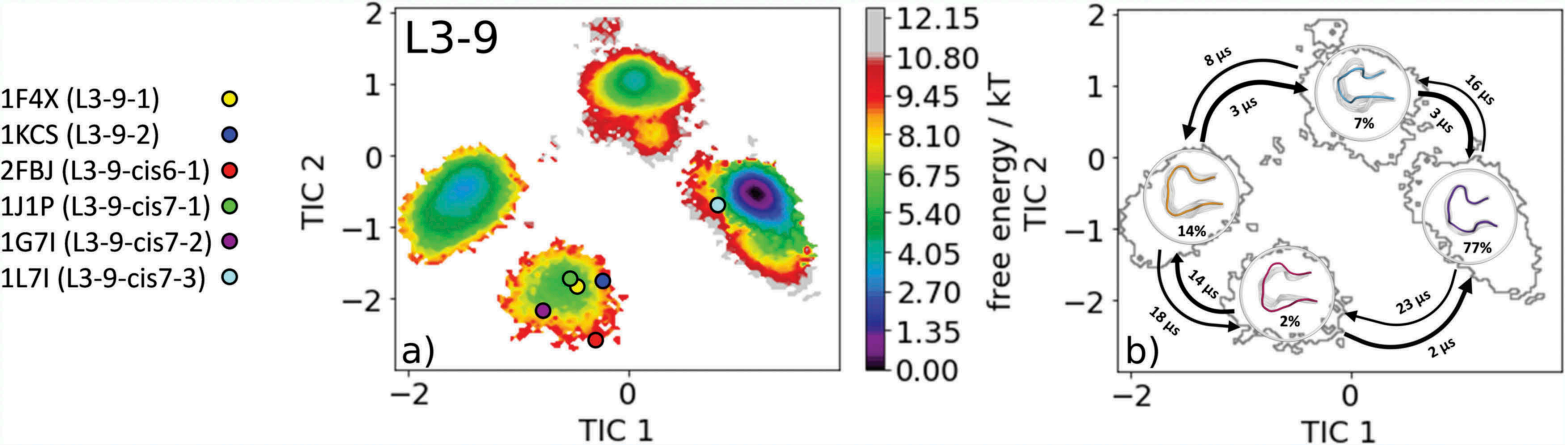
(a) Free energy surface of the CDR-L3 loop with a loop length of nine residues including the projected six available canonical cluster median representatives. The legend and color-coding of all canonical cluster representatives are shown on the left. (b) Contours of the free energy surface are displayed in the background of the Markov-state model. The macrostate representatives with the respective macrostate ensemble and transition kinetics are also included. The macrostate representatives were colored independent of the canonical cluster representatives in (a) and summarize the kinetically relevant conformations of the CDR-L3 loop in solution. We obtained four macrostates, in which all canonical cluster medians are present.

The canonical cluster transitions of the CDR-L3 loop in solution have recently been shown for four CDR-L3 loop examples with different lengths. The most common CDR-L3 loop length observed in crystal structures is nine residues. There exist six different canonical clusters with this specific loop length: L3-9-1, L3-9-2, L3-9-cis6-1, L3-9-cis7-1, L3-9-cis7-2 and L3-9-cis7-3. As the starting structure for our simulations, we chose the catalytic 28B4 Fab (PDB accession code: 1FL6), which belongs to the canonical cluster L3-9-cis7-1.^24^ Within this highest populated canonical cluster, L3-9-cis7-1 1847 crystal structures are present. Following the procedure described in the methods section, the obtained 135 cluster representatives were used as starting structures for every 100 ns molecular dynamics simulations. A tICA was performed of the obtained 13.5 µs of trajectories, and the resulting free energy landscape with the projected available canonical cluster medians of the same length is illustrated in Figure 4a. The free energy surface in Figure 4a displays four distinct minima in solution, while all canonical cluster medians are present within two macrostates. The highest populated canonical cluster representative crystal structure 1J1P lies in the same local shallow side minimum as the canonical cluster medians for the median crystal structures 1F4X, 1KCS, 2FBJ, and 1G7I. The representative median crystal structure for the L3-9-cis7-3 canonical cluster lies close to the dominant minimum in solution. No canonical cluster median crystal structures are located in the other two dominant solution minima. Figure 4b visualizes the results of the Markov-state model, which suggests the representative macrostate structures of the other two minima in solution should be included when designing and characterizing the CDR-L3 loop ensemble in solution. SI Figure 4 illustrates the overlay of all available canonical cluster median PDBs with the obtained CDR-L3 loop ensemble in solution combined with the contours of the free energy surface.

### CDR-H1 loop ensemble in solution

**Figure 5.**
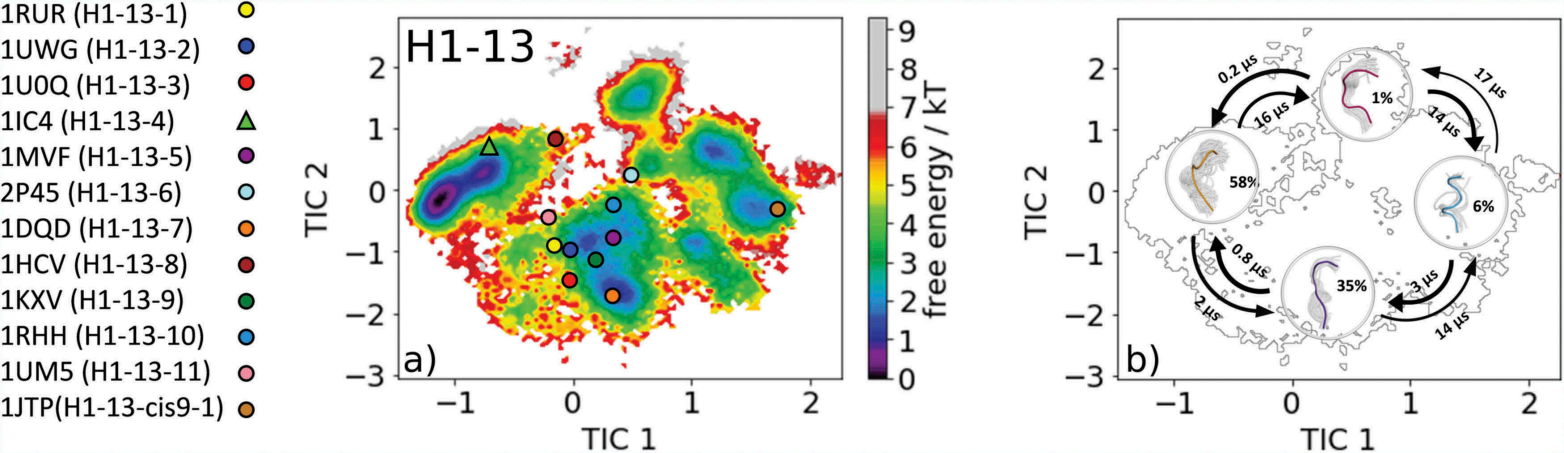
(a) Free energy surface of the CDR-H1 loop with a loop length of 13 residues including the projected 13 available canonical cluster median representatives. The legend and color-coding of all canonical cluster representatives are shown on the left. The canonical cluster representative used as starting structure for simulations is shaped as triangle, while all the other available canonical cluster median X-ray structures are visualized as circles. (b) Contours of the free energy surface are displayed in the background of the Markov-state model. The macrostate representatives with the respective macrostate ensemble and transition kinetics are also included. The macrostate representatives were colored independent of the canonical cluster representatives in (a) and summarize the kinetically relevant conformations of the CDR-H1 loop in solution. We obtained four macrostates, in which all canonical cluster medians are present.

The most common CDR-H1 loop length observed in antibody crystal structures is 13 residues. Eleven canonical clusters were classified for this specific loop length (H1-13-1, H1-13-2, H1-13-3, H1-13-4, H1-13-5, H1-13-6, H1-13-7, H1-13-8, H1-13-9, H1-13-10, H1-13-11, and H1-13-cis9-1). As starting structures for our simulations, we chose the canonical cluster H1-13-4 representative with the PDB accession code 1IC4. The canonical cluster median variable fragment (Fv) was part of a structural and thermodynamic study of the entropic contributions of salt bridge formation to the interaction between hen egg-white lysozyme (HEL) and the Fv of an anti-HEL antibody.^26^ Clustering the obtained 1 µs metadynamics simulation resulted in 130 cluster representatives that were used as starting structures for 100 ns molecular dynamics simulations. The obtained 13.0 µs molecular dynamics trajectories were used to reconstruct thermodynamics and kinetics by performing a Markov-state model. Figure 5a shows the free energy surface with the projected 12 available canonical cluster median crystal structures in the tICA space. We sampled all representative canonical cluster median PDBs, and our results revealed that nine of the 12 median structures lie in the same kinetic side minimum in solution, while the two median X-ray structures of the H1-13-4 and H1-13-8 canonical clusters lie in the global free energy minimum in solution. The representative median structure of the canonical cluster H1-13-cis9-1 was found in a local shallow side minimum separated from the other structures by contributions of the time-lagged independent component (tIC) 1. Figure 5b displays the Markov-state model of the CDR-H1 loop ensemble in solution with the highlighted macrostate representative structures and respective transition kinetics and state probabilities. The conformational transitions between the four macrostates occur in the low µs timescale. This example clearly shows, in line with the observations of the CDR-L1, CDR-L2, and CDR-L3 ensembles in solution, that one CDR-H1 loop sequence can adopt various conformations in solution. We observed conformational transitions between different canonical structures independent of the length and assigned canonical cluster, and therefore the CDR-H1 loop needs to be described as a conformational ensemble in solution.

**Figure 6.**
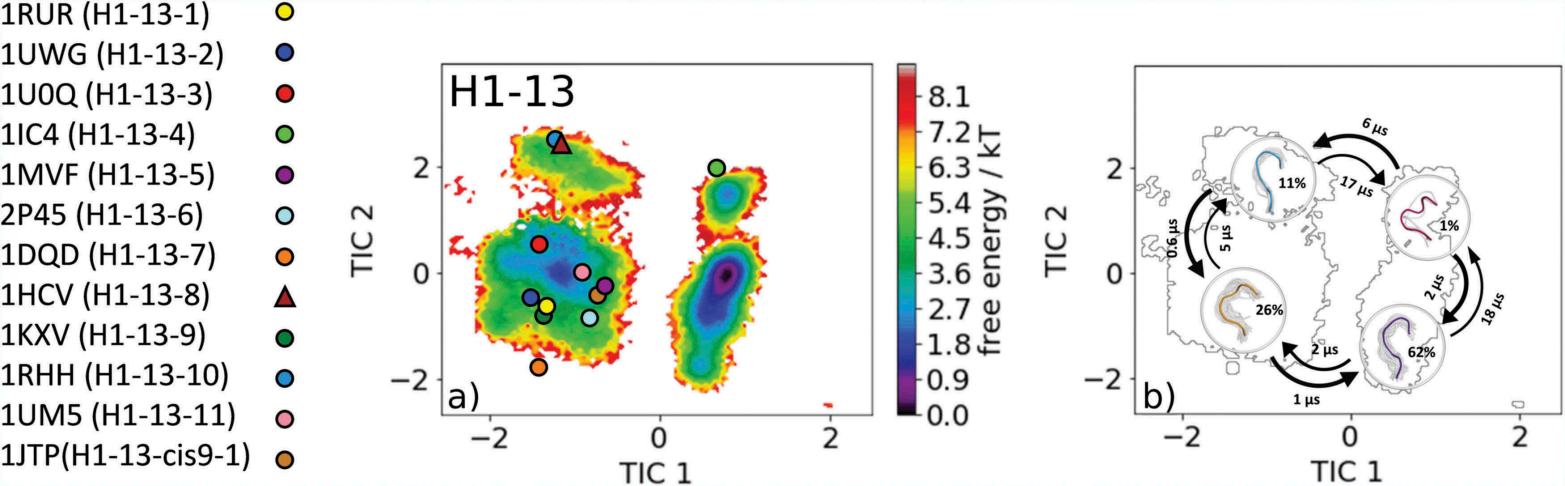
(a) Free energy surface of the CDR-H1 loop with a loop length of 13 residues including the projected 13 available canonical cluster median representatives. The legend and color-coding of all canonical cluster representatives are shown on the left. The canonical cluster representative used as starting structure for simulations is shaped as triangle, while all the other available canonical cluster median X-ray structures are visualized as circles. (b) Contours of the free energy surface are displayed in the background of the Markov-state model. The macrostate representatives with the respective macrostate ensemble and transition kinetics are also included. The macrostate representatives were colored independent of the canonical cluster representatives in (a) and summarize the kinetically relevant conformations of the CDR-H1 loop in solution. We obtained four macrostates, in which 12 of 13 canonical cluster medians are present. We also identify a potentially relevant conformation in solution.

Due to the fact that the majority of available antibody X-ray structures reveal a CDR-H1 loop length of 13 residues, we decided to include the results obtained for the simulations of the H1-13-8 canonical cluster representative (PDB accession code: 1HCV) as well. As described in the methods section, the obtained 1 µs metadynamics trajectory of the llama heavy chain variable domain against the alpha subunit of human chronic gonadotropin was clustered, and the resulting 130 clusters were used as starting structure for molecular dynamics simulations.^27^ The free energy surface of the 13.0 µs molecular dynamics trajectories with the projected 12 canonical cluster median representatives is shown in Figure 6a. Eight of the 12 canonical cluster median structures lie in the same local minimum in solution, while the representative median X-ray structure of the H1-13-7 (PDB code: 1DQD) cluster was not sampled. The canonical cluster representatives of the H1-13-8 (PDB code: 1HCV) and H1-13-10 (PDB code: 1RHH) lie in the same local shallow side minimum. The median crystal structure of the H1-13-4 cluster (PDB code: 1IC4) is present in another local side minimum. The summary and overlay of the canonical cluster median crystal structures with the observed ensemble in solution and the contours of the tICA free energy in the background are shown in SI Figure S6. Surprisingly, no canonical structure can be found in the global minimum, and this result strongly indicates that the macrostate representative of the dominant free energy minimum in solution has to be included when characterizing the CDR-H1 loop ensemble. Figure 6b presents the results of the Markov-state model and shows the obtained four macrostate representative structures with the respective ensemble in the background. The transitions occur in the low µs timescale and the state probabilities are represented by the broadness of the ensemble.

### CDR-H2 loop ensemble in solution

**Figure 7.**
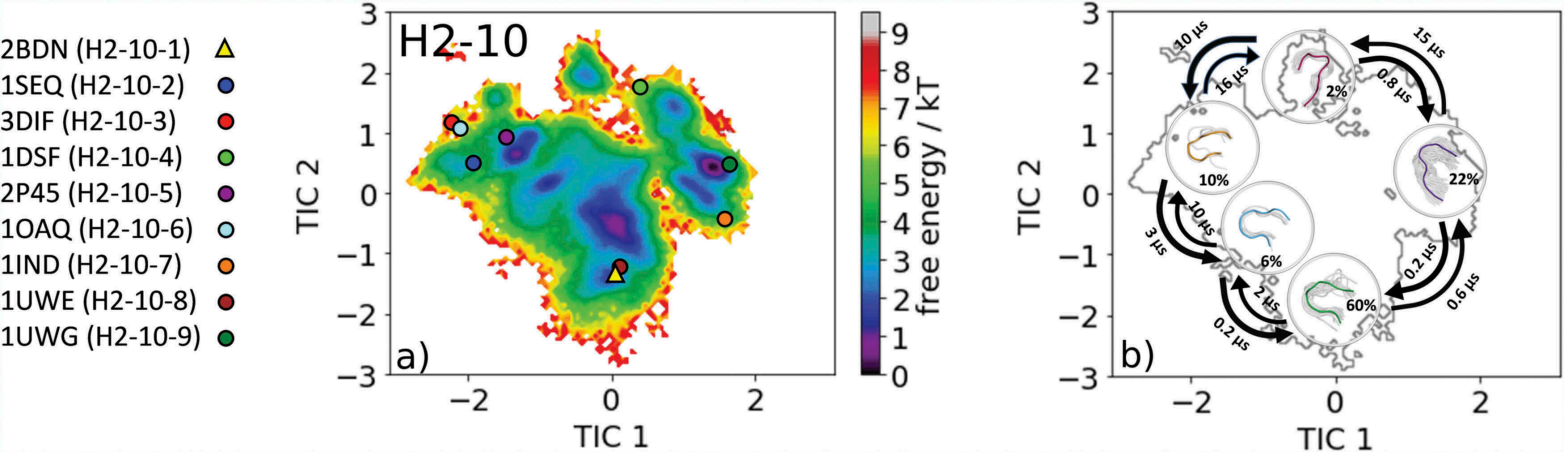
(a) Free energy surface of the CDR-H2 loop with a loop length of 10 residues including the projected available canonical cluster median representatives. The legend and color-coding of all canonical cluster representatives are shown on the left. The canonical cluster representative used as starting structure for simulations is shaped as triangle, while all the other available canonical cluster median X-ray structures are visualized as circles. (b) Contours of the free energy surface are displayed in the background of the Markov-state model. The macrostate representatives with the respective macrostate ensemble and transition kinetics are also included. The macrostate representatives were colored independent of the canonical cluster representatives in (a) and summarize the kinetically relevant conformations of the CDR-H2 loop in solution. We obtained four macrostates, in which all canonical cluster medians are present.

The most common CDR-H2 loop length observed in crystal structures is 10 residues. With this specific loop length, nine canonical clusters (H2-10-1, H2-10-2, H2-10-3, H2-10-4, H2-10-5, H2-10-6, H2-10-7, H2-10-8 and H2-10-9) were classified. To characterize the CDR-H2 loop in solution, we chose the canonical cluster median crystal structure of the highest populated CDR-H2 loop canonical cluster H2-10-1 with the PDB accession code 2BDN. Within this canonical cluster, 1521 crystal structures are present. 11K2 (PDB code: 2BDN) is a blocking antibody and shows activity against several human and murine monocyte chemoattractant proteins.^28^ Following the procedure described in the methods section, we obtained 215 cluster representatives and, after simulating each representative for 100 ns, we used the resulting 21.5 µs of trajectories to calculate a Markov-state model to reconstruct thermodynamics and kinetics. The free energy surface with the projected canonical cluster representatives is shown in Figure 7a. All nine canonical cluster representatives were present within our CDR-H2 loop in solution. The Markov-state model revealed five macrostates and the results are illustrated in Figure 7b. Within the global free energy minimum in solution two canonical cluster median crystal structures of the H2-10-1 (PDB code: 2BDN) and the H2-10-3 (PDB code: 3DIF) were present. In line with the observations of the previous examples, we see that various canonical cluster median structures belong to the same kinetic minimum in solution, and thus might be combined. Figure 7b displays the macrostate representative structures with the respective CDR-H2 loop ensemble in solution, and again the broadness of the ensemble in the background reflects the state probability.

**Figure 8.**
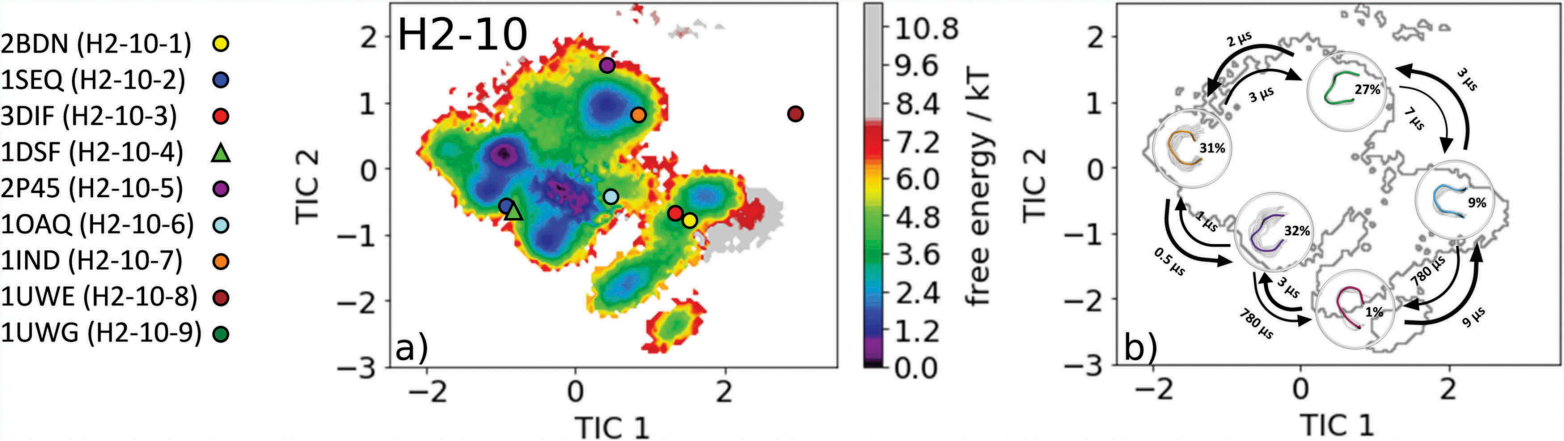
(a) Free energy surface of the CDR-H2 loop with a loop length of ten residues including the projected available canonical cluster median representatives. The legend and color-coding of all canonical cluster representatives are shown on the left. The canonical cluster representative used as starting structure for simulations is shaped as triangle, while all the other available canonical cluster median X-ray structures are visualized as circles. (b) Contours of the free energy surface are displayed in the background of the Markov-state model. The macrostate representatives with the respective macrostate ensemble and transition kinetics are also included. The macrostate representatives were colored independent of the canonical cluster representatives in (a) and summarize the kinetically relevant conformations of the CDR-H2 loop in solution. We obtained four macrostates, in which eight of nine canonical cluster medians are present.

The overlay of the canonical clusters belonging to the same kinetic minimum are shown in SI Figure S7 combined with the contours of the free energy landscape in the background. In addition, the median crystal structure representative of the H2-10-4 canonical cluster 1DSF, which is a disulfide-stabilized anti-cancer antibody,^29^ was also simulated for 1 µs metadynamics simulation and each of the resulting 109 clusters were simulated for 100 ns. For the obtained 10.9 µs of trajectories, we performed tICA combined with a Markov-state model and the results are shown in Figure 8. Figure 8a shows the free energy landscape with the projected canonical clusters. Besides the median PDB of the H2-10-8 canonical cluster, all median representatives were present within the ensemble in solution. Again, as in the previous example, the representatives of the H2-10-1 and the H2-10-3 canonical clusters (2BDN and 3DIF, respectively) lie in the same kinetic side minimum. The Markov-state model revealed five macrostates with fast transition kinetics between the canonical cluster representatives and ensembles in solution (Figure 8b and SI Figure S8). The macrostate representatives with transition timescales are visualized in Figure 8b with the respective macrostate CDR-H2 loop ensemble in solution in the background. The transitions between the different canonical clusters occur in the low µs timescale and emphasize that for a given CDR-H2 loop sequence several canonical structures have to be considered.

### CDR-H3 loop ensemble in solution

**Figure 9.**
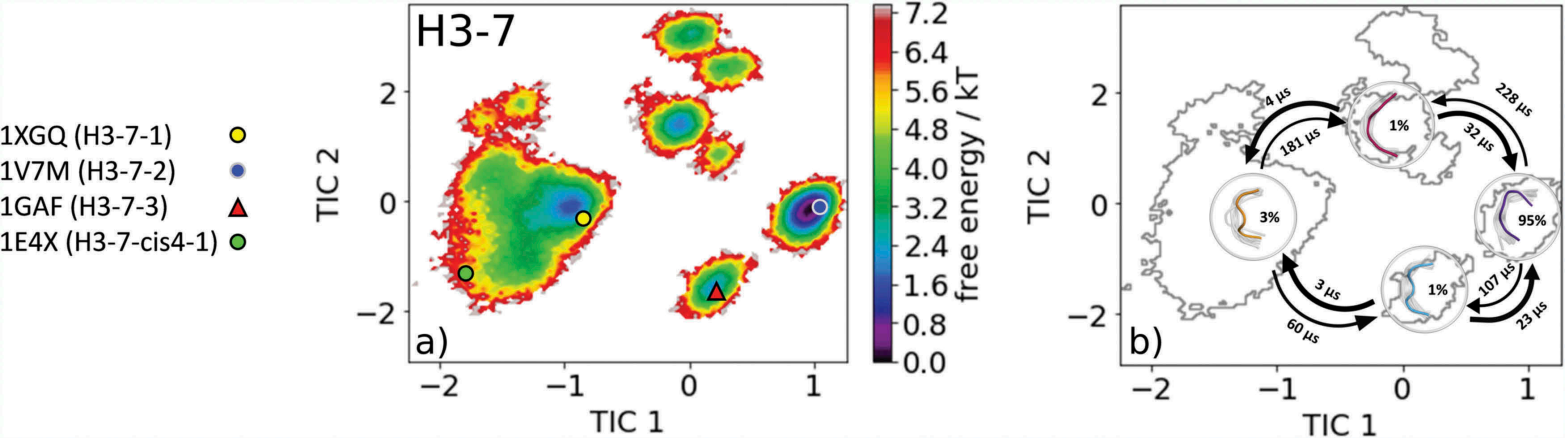
(a) Free energy surface of the CDR-H3 loop with a loop length of seven residues including the projected available canonical cluster median representatives. The legend and color-coding of all canonical cluster representatives are shown on the left. The canonical cluster representative used as starting structure for simulations is shaped as triangle, while all the other available canonical cluster median X-ray structures are visualized as circles. (b) Contours of the free energy surface are displayed in the background of the Markov-state model. The macrostate representatives with the respective macrostate ensemble and transition kinetics are also included. The macrostate representatives were colored independent of the canonical cluster representatives in (a) and summarize the kinetically relevant conformations of the CDR-H3 loop in solution. We obtained four macrostates, in which all canonical cluster medians are present.

Characterizing the CDR-H3 loop has been a major challenge in antibody design because it does not fit into the description of canonical structure models. Various studies focused on classifying and characterizing the CDR-H3 loop to improve the quality of antibody structures.^20,30,31^ The most common CDR-H3 loop length that was classified into canonical clusters is seven residues. With this specific loop length, there exist four canonical clusters H3-7-1, H3-7-2, H3-7-3, and H3-7-cis4-1. We chose the representative canonical cluster median of the H3-7-3 canonical cluster (PDB accession code 1GAF) starting structure for 1 µs metadynamics simulation. This 48G7 hybridoma line Fab, binding to the hapten 5-(para-nitrophenylphsponate)-pentanoic acid, was derived from the germline esterolytic antibody fragments with the PDB codes 2RCS and 1AJ7.^32^ Clustering of the 1 µs metadynamics simulation resulted in 120 cluster representatives that were simulated for each 100 ns. The resulting 12.0 µs of trajectories were used to reconstruct thermodynamics and kinetics of the obtained CDR-H3 loop ensemble in solution (Figure 9). Figure 9a shows the resulting free energy landscape, including the projection of the canonical cluster median crystal structures. All four available crystal structures were present in the CDR-H3 loop ensemble in solution, and the representative structure of the H3-7-2 canonical cluster lies in the global minimum in solution. The representatives of the H3-7-1 and H3-7-cis4-1 canonical clusters (PDB codes: 1XGQ and 1E4X) belong to the same kinetic minimum, while the H3-7-3 (PDB code: 1GAF) belongs to another kinetic local side minimum. The Markov-state model in Figure 9b revealed three macrostates, in which all four canonical cluster median structures were sampled. However, apart from sampling all four canonical median structures, another kinetically relevant state in solution was obtained, which has to be considered. The four macrostate representative structures with the respective macrostate ensemble in the background, reflecting the state probabilities, and the transition timescales between the macrostates are shown in Figure 9b. Again, SI Figure S9 visualizes the canonical cluster median structures combined with the obtained ensemble in solution with the free energy contours in the background.

**Figure 10.**
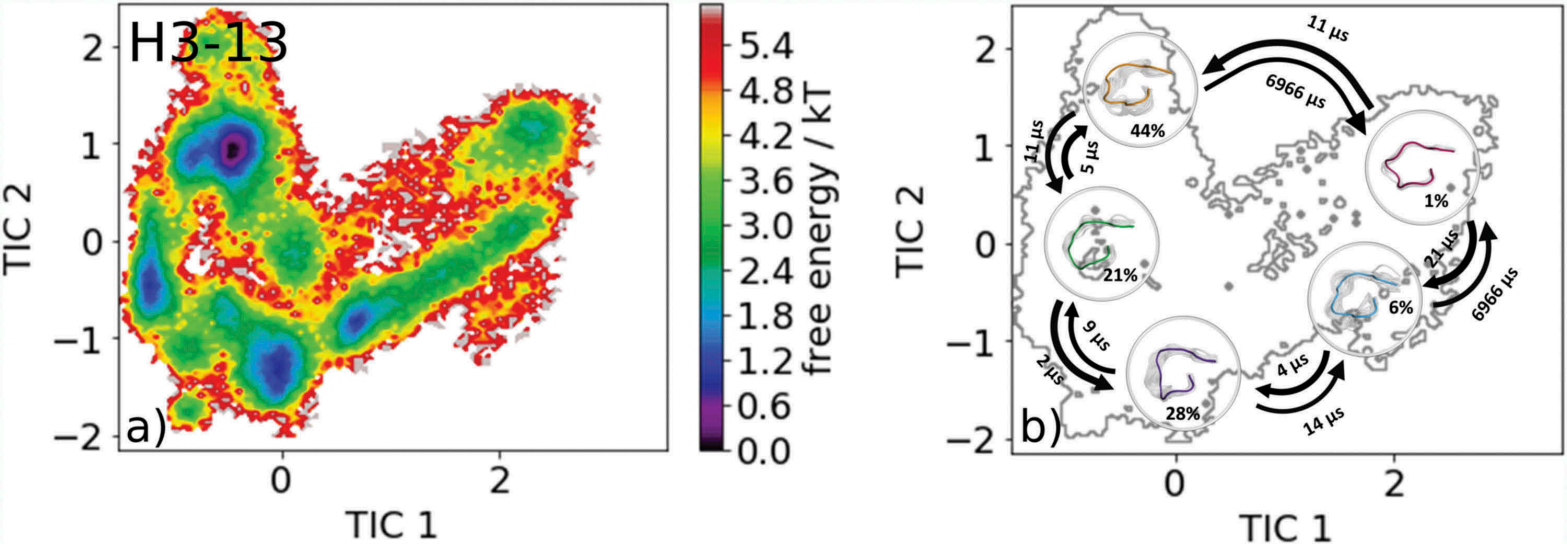
(a) Free energy surface of the CDR-H3 loop with a loop length of 13 residues. As for this specific loop length, no canonical cluster could be assigned no projections of X-ray structures are shown. (b) Contours of the free energy surface are displayed in the background of the Markov-state model. The macrostate representatives with the respective macrostate ensemble and transition kinetics are also included. The macrostate representatives summarize the kinetically relevant conformations of the CDR-H3 loop in solution. We obtained five macrostates and identified potentially relevant solution structures.

Besides presenting possible CDR-H3 loop canonical cluster transitions, we also show an example of a CDR-H3 loop with length 13 without any canonical cluster assignment. As the starting structure, we chose the crystal structure of the AL-57 antibody binding to integrin lymphocyte function-associated antigen-1 with the PDB accession code 3HI6.^33^ Figure 10 shows the results of the obtained 8.3 µs of molecular dynamics simulations. The free energy surface is illustrated in Figure 10a and the corresponding Markov-state model with the macrostate representatives, transition kinetics and state probabilities are visualized in Figure 10b.

The root-mean-square deviation values and the dihedral angle distances used by North et al.^13^ for the canonical cluster centroids for all CDR-loops to the closest neighbor in our molecular dynamics simulations are provided in SI Table 1.

## Discussion

In this study, we characterized the structural diversity of all CDR loops in solution and provide a kinetic and thermodynamic analysis of the conformational space supported by strong experimental structural information. We also investigated the conformational transitions between canonical clusters of all CDR loops. The structural description of the CDR-H3 and CDR-L3 loops is known to be a major hurdle in antibody design because they show the greatest diversity in length and sequence and are located in the center of the binding site.^34^ Following the concept of conformational diversity, i.e., that the same antibody can adopt various conformations, some studies suggested that this conformational diversity could also increase the functional diversity of a limited repertoire of sequences, and thereby facilitate the evolution of new antibodies.^35–39^ The sequence and structural classification of antibody CDR loops has been the focus of various studies and is still an evolving process due to the increasing number of antibody Fab structures.^5,11,13,17,40,41^ The canonical clusters of the CDR loops are diverse in length and sequence composition, and with increasing CDR loop length, their population and predictive value decrease.^20^ Additionally, it has been shown for CDR-H3 and CDR-L3 loop ensembles that they should be described as conformational ensembles in solution.^21,22,30^ Also, special care has to be taken when analyzing CDR loops by Fab crystal structures because of distortions due to crystal packing effects.^22,42^

Our data, shown in Figure 1a, confirm these observations for the CDR-L1 loop ensemble in solution. The representatives of the L1-11-1 and L1-11-2 canonical clusters 2D7 T and 1ZAN, which were crystallized without antigen, lie in the same kinetic shallow side minimum, while the representative of the L1-11-3 canonical cluster 1W72, crystallized with antigen, belongs to the global free energy minimum in solution. This observation is in line with the very similar sequence profiles of the L1-11-1 and the L1-11-2 canonical clusters, while the canonical cluster L1-11-3 entirely contains lambda light chains. This might also explain the low probability and high energy of the L1-11-1 and the L1-11-2 canonical cluster conformations when starting from the L1-11-3 cluster representative. Furthermore, for the CDR-L1 loop, we were able to identify additional dominant minima (Figure 1a), which could be included when characterizing the CDR-L1 loop ensemble in solution. Figure 1b and SI Figure 1 reveal the transition kinetics between different canonical cluster structures and identify dominant solution structures, including the respective state probabilities.

SI Figure S10 and SI Figure S11 show the free energy landscapes of the simulations started from the L1-11-1 (17.4 µs of trajectories) and L1-11-2 (24.4 µs of trajectories) canonical cluster representatives with the PDB accession codes 1ZAN and 2D7 T, respectively. We observe transitions between all three available canonical clusters and observe in these two examples that, when starting from the kappa light chain, the canonical cluster representative of cluster L1-11-3 lies in a local side minimum.

The comparison of the canonical cluster median with the respective macrostate ensemble is illustrated in SI Figure S12 and S13 with the tICA contours in the background. In line with the results of the CDR-L1 loop, we observe for the CDR-L2 loop (eight residues) in Figure 2a that within the global free energy minimum in solution three canonical structures were present and the highest populated canonical cluster representative 1YEJ, which was crystallized with antigen, lies directly in the dominant solution minimum. The Markov-state model in Figure 2b reveals that two macrostates within four of five canonical cluster representatives were sampled.

Figure 3a shows for a CDR-L2 loop with a length of 12 residues that, besides sampling the two available canonical cluster representatives, we identified two additional potentially relevant conformations of the CDR-L2 loop ensemble in solution. Figure 3b illustrates the results of the Markov-state model and displays the dominant ensembles in solution for the respective macrostates including transition kinetics.

Characterization of the CDR-L3 loop in solution is shown in Figure 4a. Antibodies with the CDR-L3 loop length of nine residues represent the most common and highest populated CDR-L3 loop.^12,13,20,40,43^ Within our CDR-L3 loop ensemble in solution, we identified five canonical cluster representatives in the same kinetic side minimum, while the sixth available canonical cluster median of the L3-9-cis7-3 was close to the dominant minimum in solution. Besides the two macrostates, in which all canonical clusters were present, two additional potentially relevant CDR-L3 loop conformations in solution could be identified. The macrostate representatives with the respective macrostate ensemble including the transition kinetics are shown in Figure 4b. Very similar findings were observed for the CDR-H1 loop examples in Figures 5 and 6. For the canonical cluster H1-13-4 representative 1IC4, we were able to observe all available representative canonical cluster structures and identified that within one kinetic minimum in solution 10 of the 13 available canonical cluster median structures were present and might be combined (Figure 5). Within the dominant minimum in solution, one representative of the H1-13-4 canonical cluster could be identified, while the median structure for the H1-13-8 canonical cluster is situated in a very unfavorable region of this minimum. The representative median crystal structure of the H1-13-8 canonical cluster was crystallized without antigen, same as the representative of the highest populated canonical cluster H1-13-1 (PDB code: 1RUR) and the representatives of the H1-13-3, H1-13-5 and H1-13-7 canonical clusters. The majority of these CDR-H1 loop structures crystallized without antigen belong to the same minimum but lie in unfavorable regions of the free energy surface. This effect could be explained due to crystal packing effects in the unit cell which lead to a distortion of the CDR-H1 loop.

The results of the second CDR-H1 loop example (Figure 6) started from the representative of the H1-13-4 canonical cluster again reveal various conformational transitions between different canonical structures. Within the four obtained macrostates, 12 of 13 canonical cluster median structures are present, but eight canonical cluster structures actually belong to the same kinetic minimum and might be combined. Especially the canonical cluster representatives of the clusters H1-13-1 and H1-13-4 are very similar to each other and structurally related to each other by a peptide flip at the positions 8 and 9 (i.e., one residue in psi is flipped by 180° and the next residue is flipped in phi by 180°). Figure 6b visualizes the macrostate representative structures with the respective ensemble in the background including the transition kinetics and state probabilities. The broadness of the ensemble reflects also the population of the respective macrostate. Surprisingly, no canonical cluster representative could be identified within the dominant free energy minimum, indicating the existence of other more probable and dominant CDR-H1 loop conformations in solution. The transitions between the four macrostates of this antibody occur in the nano-to-microsecond timescale, in which we again observed transitions between canonical clusters.

In line with all previously discussed CDR loops, the CDR-H2 loop conformational ensemble in solution (Figure 7) reveals for the highest populated CDR-H2 canonical cluster H2-10-1 (PDB code: 2BDN) that all nine canonical cluster representatives were present within the ensemble in solution. The highest populated canonical cluster median H2-10-1 belongs together with the H2-10-8 canonical cluster structure to the global minimum in solution. The Markov-state model in Figure 7b illustrates the kinetically defined macrostate representatives and characterizes the transition kinetics and state probabilities. Again, in line with the previous results, we were able to sample various transitions between canonical cluster representatives in the microsecond timescale.

Besides kinetically profiling the available canonical cluster representatives, we identified another potentially relevant CDR-H2 loop solution structure. The results of the second analyzed CDR-H2 loop X-ray structure of the H2-10-4 canonical cluster are shown in Figure 8 and eight of nine available canonical cluster representatives are present within the CDR-H2 loop ensemble in solution. The Markov-state model in Figure 8b reconstructs the kinetics and thermodynamics of the CDR-H2 loop ensemble in solution and allows the combination of kinetically equivalent canonical clusters. The structural characterization of the CDR-H3 loop has already been subject of numerous studies, and the structure prediction and classification of this highly flexible CDR loop has been challenging.^20,31,44^

The clustering of available CDR-H3 loop conformations with short CDR-H3 loop lengths (maximum nine residues) was of high predictive value.^20^ Therefore, we chose the representative of the H3-7-3 canonical cluster (PDB code: 1GAF) to characterize the conformational ensemble in solution. For this specific loop length, four possible canonical clusters exist. Figure 9a displays the free energy surface and shows that within our CDR-H3 loop ensemble in solution all available canonical cluster median X-ray structures are present. The Markov-state model in Figure 9b visualizes the representative macrostates and the transition timescales of the distinct CDR-H3 loop conformations in the microsecond timescale.

Besides sampling all canonical cluster representatives, we identified another kinetically distinct structure as a potentially relevant solution structure. Figure 10 illustrates the results of an antibody CDR-H3 loop of the length of 13, where no canonical cluster could be assigned because of the decrease in reliability of the canonical clusters with increasing loop length.^20^ The free energy surface in combination with the Markov-state model, illustrated in Figure 10, reveals five macrostates and shows the respective transition kinetics in the microsecond timescale. Within the highest populated macrostate, the binding competent conformation is present, and this result confirms the observations that within the preexisting ensemble of conformations the CDR-H3 loop conformation is optimized to bind the antigen.^22^ Figure 10b illustrates representative macrostate structures, being potentially relevant CDR-H3 loop solution structures.

For structure design, our results clearly show that for a given CDR loop sequence various conformations, including different canonical cluster representatives, have to be considered.

In conclusion, we characterized the ensemble in solution for all CDR loops with different loop lengths and were able to structurally, kinetically and thermodynamically profile the respective CDR loop conformational space. Supported by strong experimental structural information, we observed canonical cluster transitions in the micro-to-millisecond timescale and identified additional dominant solution structures that should be considered in the antibody structure design. Besides sampling the majority of the available canonical cluster representatives, our results revealed that various canonical cluster median X-ray structures belong to the same kinetic minimum in solution, and thus might be combined. These findings have implications in the field of antibody structure design and extend the models of static canonical clusters to a description of all CDR loops as conformational ensemble in solution.

## Methods

A previously published method characterizing the CDR-H3 and CDR-L3 loop ensemble in solution^22,30^ was used to investigate the conformational diversity of all CDR loops. Experimental structure information was available for all considered antibody fragments. The starting structures for simulations were prepared in MOE (Molecular Operating Environment, Chemical Computing Group, version 2018.01) using the Protonate3D tool.^45,46^ To neutralize the charges we used the uniform background charge.^47–49^ Using the tleap tool of the AmberTools16^47,48^ package, the crystal structures were soaked with cubic water boxes of TIP3P water molecules with a minimum wall distance of 10 Å to the protein.^50^ For all crystal structures parameters of the AMBER force field, 14SB were used.^51^ The antibody fragments were carefully equilibrated using a multistep equilibration protocol.^52^

### Metadynamics simulations

To enhance the sampling of the conformational space, well-tempered metadynamics^53–55^ simulations were performed in GROMACS^56,57^ with the PLUMED 2 implementation.^58^ As collective variables, we used a linear combination of sine and cosine of the ψ torsion angles of the CDR loops calculated with functions MATHEVAL and COMBINE implemented in PLUMED 2.^58^ As discussed previously, the ψ torsion angle captures conformational transitions comprehensively.^38,39^ We decided to include the ψ torsion angles of a neighboring CDR loop because we observed an improved sampling efficiency.^59^ The simulations were performed at 300 K in an NpT ensemble. We used a Gaussian height of 10.0 kcal/mol. Gaussian deposition occurred every 1000 steps and a bias factor of 10 was used. One µs metadynamics simulations were performed for each available antibody fragment crystal structure. The resulting trajectories were clustered in cpptraj^48,60^ by using the average linkage hierarchical clustering algorithm with a distance cutoff criterion of 1.2 Å resulting in a large number of clusters. The cluster representatives for the antibody fragments were equilibrated and simulated for 100 ns using the AMBER16^47^ simulation package.

### Molecular dynamics simulations

Molecular dynamics simulations were performed in an NpT ensemble using pmemd.cuda.^61^ Bonds involving hydrogen atoms were restrained by applying the SHAKE algorithm,^62^ allowing a time step of 2.0 fs. Atmospheric pressure of the system was preserved by weak coupling to an external bath using the Berendsen algorithm.^63^ The Langevin thermostat^64^ was used to maintain the temperature during simulations at 300 K.

With the obtained trajectories, we performed a tICA using the python library PyEMMA 2 employing a lag time of 10 ns.^65^ Thermodynamics and kinetics were calculated with a Markov-state model^66^ by using PyEMMA 2, which uses the k-means clustering algorithm^67^ to define microstates and the Perron cluster analysis (PCCA+) clustering algorithm^68^ to coarse grain the microstates to macrostates. The sampling efficiency and the reliability of the Markov-state model (e.g., defining optimal feature mappings) can be evaluated with the Chapman–Kolmogorov test^69,70^ by using the variational approach for Markov processes^71^ and by taking into account the fraction of states used, as the network states must be fully connected to calculate probabilities of transitions and the relative equilibrium probabilities. To build the Markov-state model, we used the backbone torsions of the respective CDR loop, defined 150 microstates using the k-means clustering algorithm and applied a lag time of 10 ns. Depending on the CDR loop and loop length, different numbers of canonical clusters were available and the median crystal structure information for each CDR loop length was extracted from the PyIgClassify database^13^ and compared to the obtained CDR loop ensemble in solution.

## Supplementary Material

Supplemental Material
