# Supplementary material for: Antibody CDR loops as ensembles in solution vs. canonical clusters from X-ray structures
Source: MAbs. 2020 Apr 7;12(1):1744328. doi: 10.1080/19420862.2020.1744328 (PMC7153821; doi:10.1080/19420862.2020.1744328)
Supplement: Supplemental Material [file kmab-12-01-1744328-s001.docx]

Supplemental Information


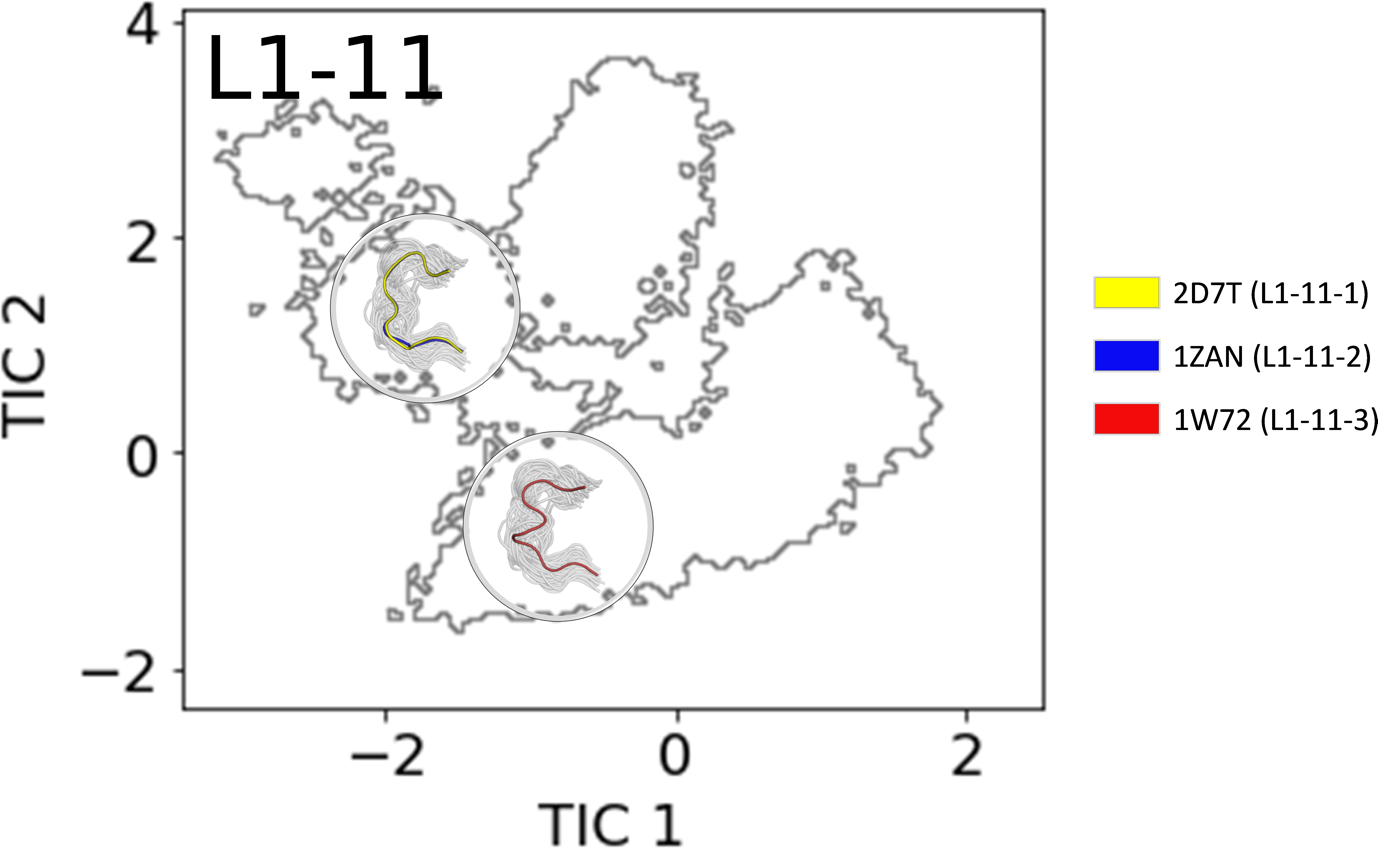


SI Figure S1: Contours of the tICA free energy surface of the CDR-L1 loop with the projected canonical cluster median crystal structures and the representative CDR-L1 loop ensemble in the background. On the right the color-coding of the canonical cluster medians is shown.


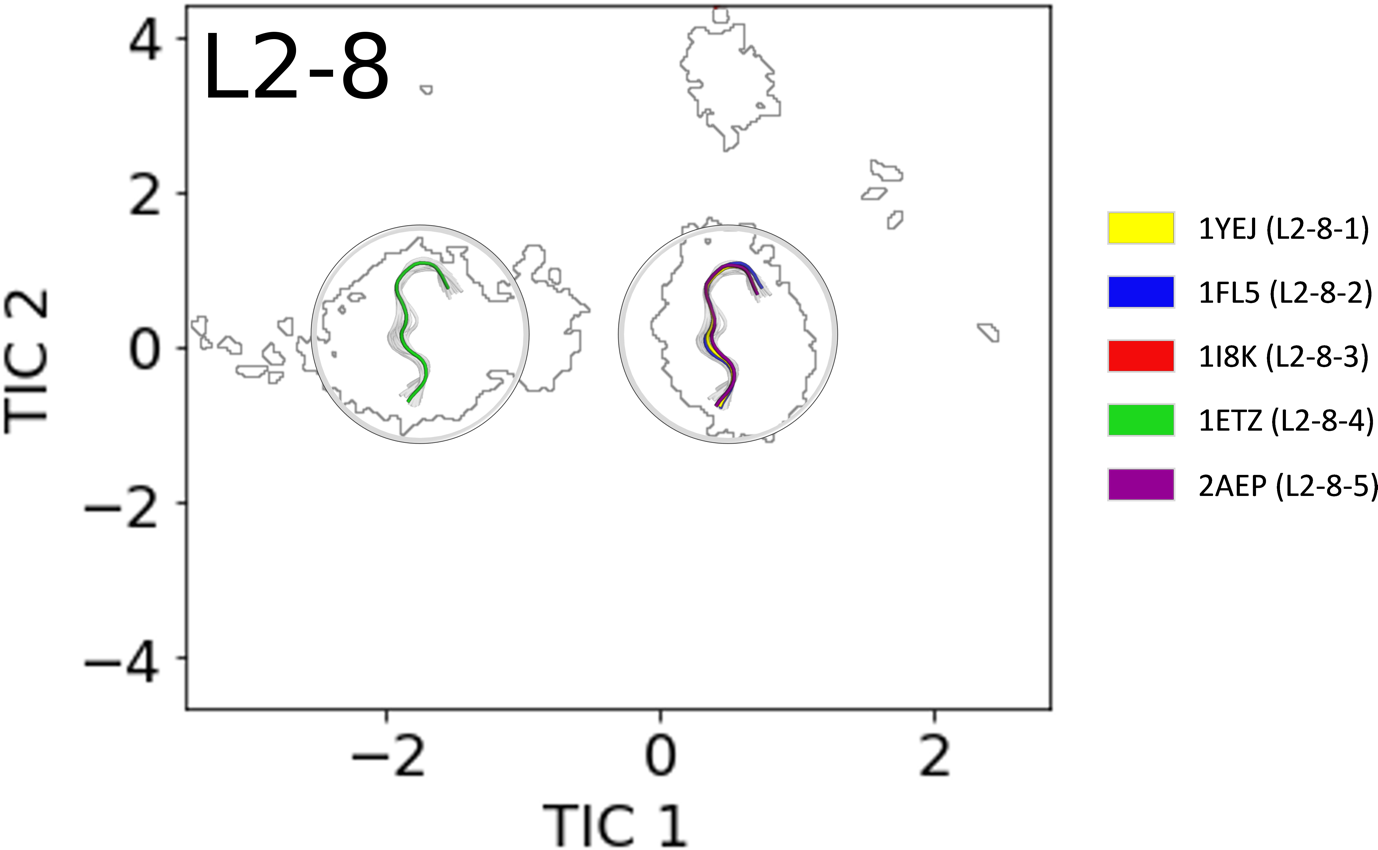


SI Figure S2: Contours of the tICA free energy surface of the CDR-L2 loop with the projected canonical cluster median crystal structures and the representative CDR-L2 loop ensemble in the background. On the right the color-coding of the canonical cluster medians is shown.


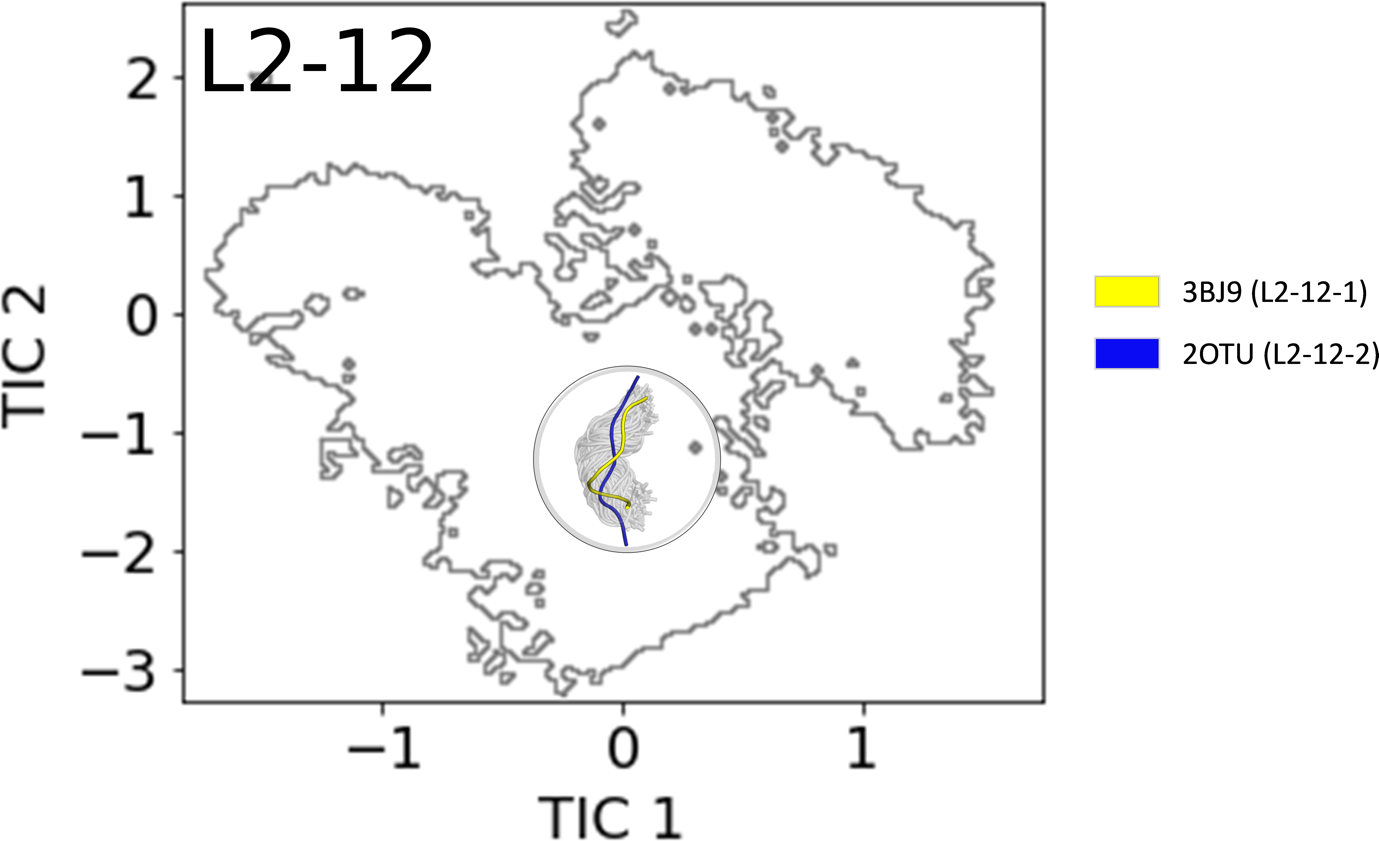


SI Figure S3: Contours of the tICA free energy surface of the CDR-L2 loop with the projected canonical cluster median crystal structures and the representative CDR-L2 loop ensemble in the background. On the right the color-coding of the canonical cluster medians is shown.


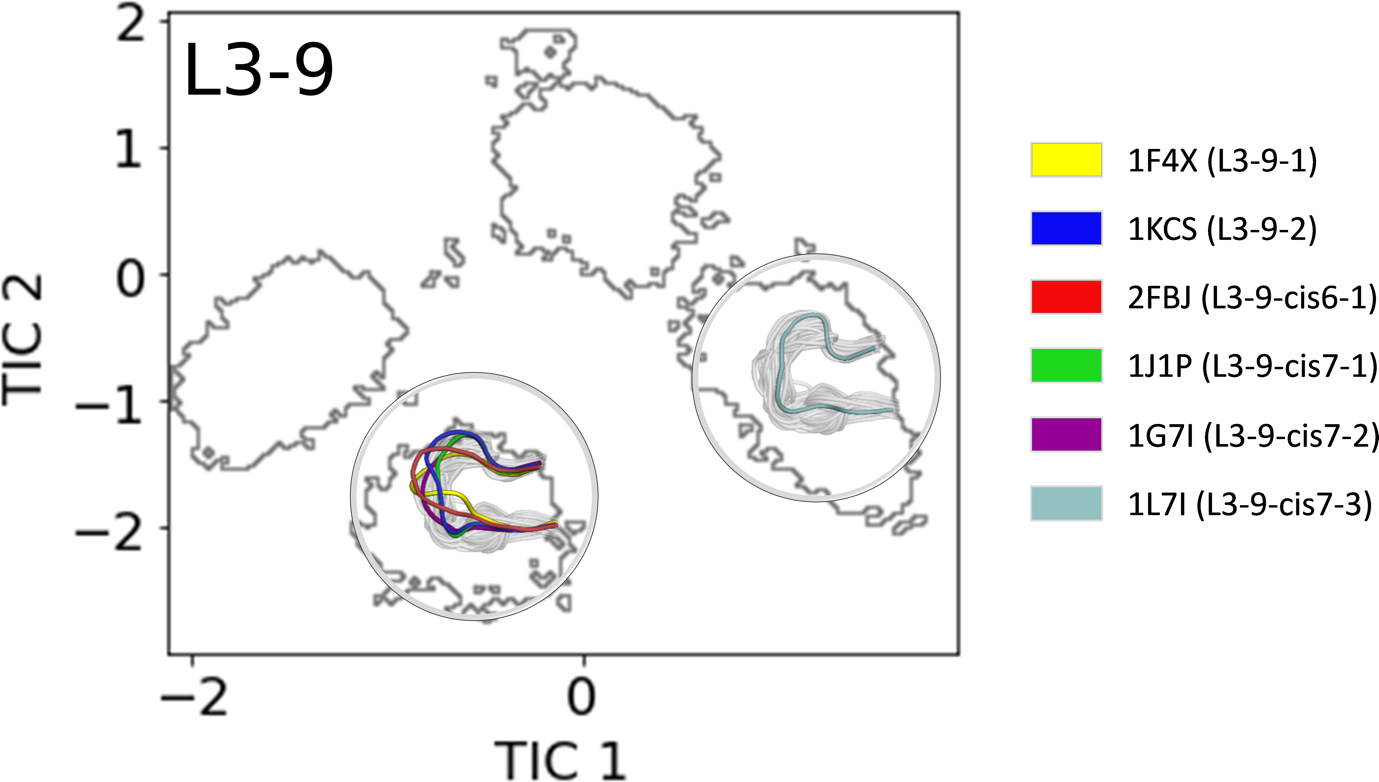


Figure 4: Contours of the tICA free energy surface of the CDR-L3 loop with the projected canonical cluster median crystal structures and the representative CDR-L3 loop ensemble in the background. On the right the color-coding of the canonical cluster medians is shown.


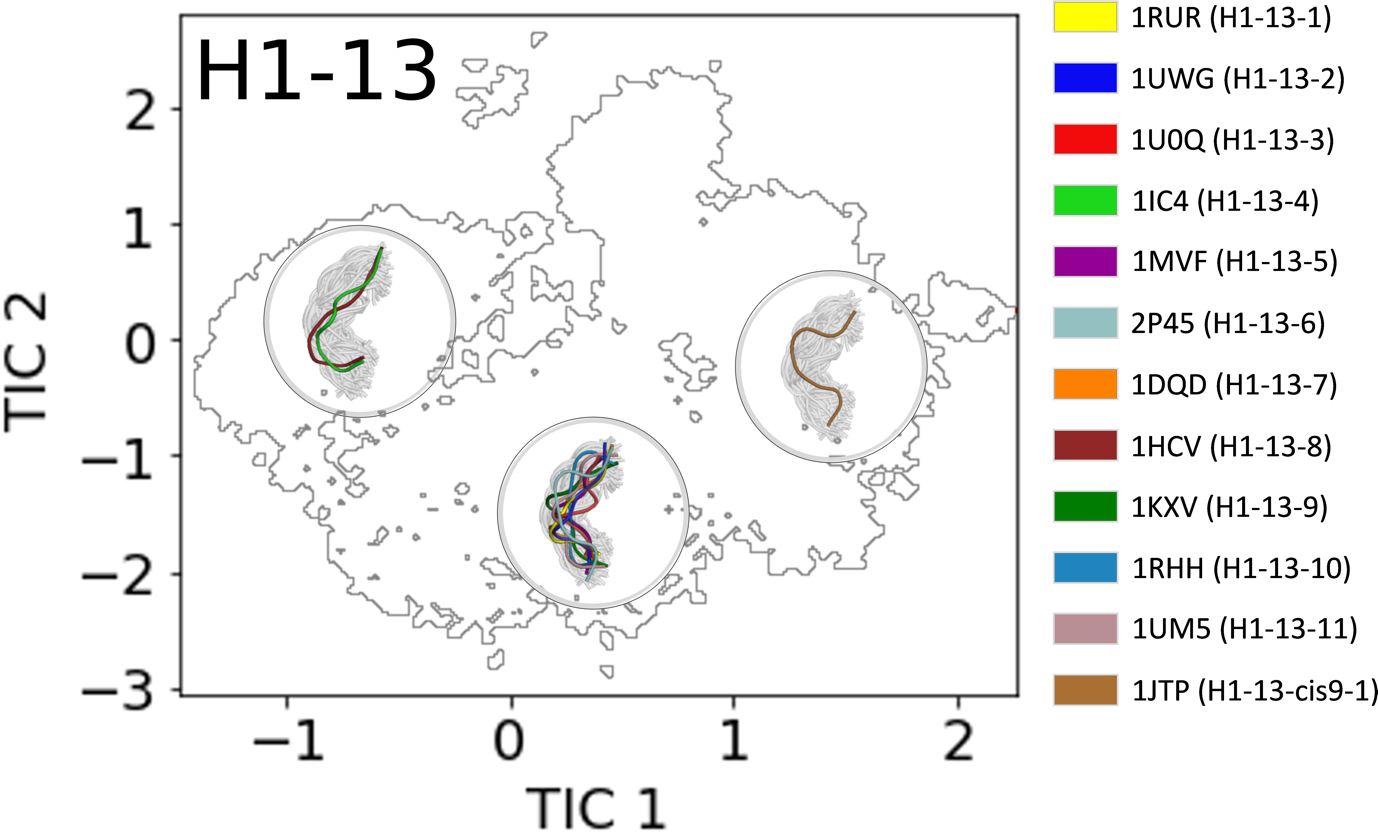


SI Figure S5: Contours of the tICA free energy surface of the CDR-H1 loop with the projected canonical cluster median crystal structures and the representative CDR-H1 loop ensemble in the background. On the right the color-coding of the canonical cluster medians is shown.


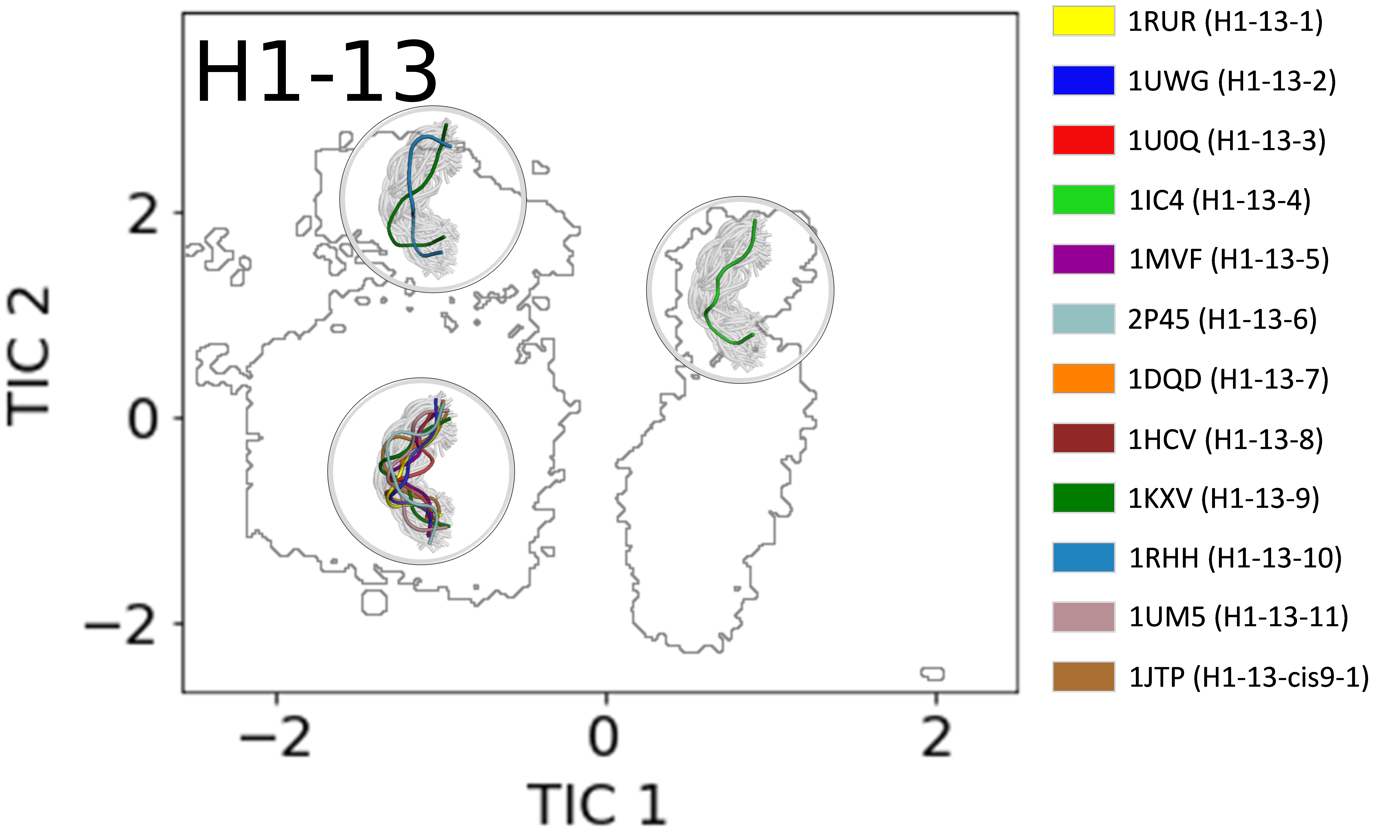


SI Figure S6: Contours of the tICA free energy surface of the CDR-H1 loop with the projected canonical cluster median crystal structures and the representative CDR-H1 loop ensemble in the background. On the right the color-coding of the canonical cluster medians is shown.


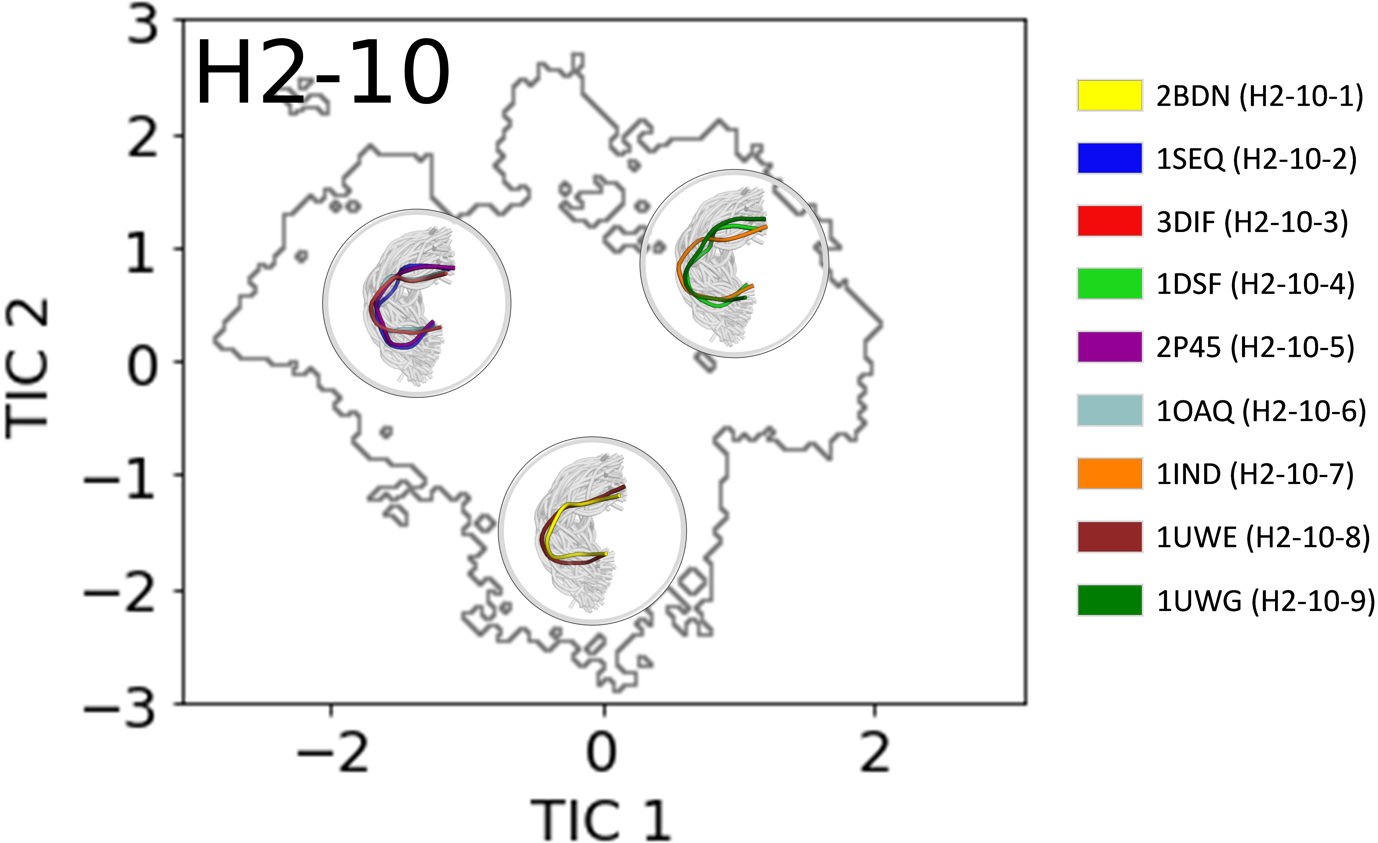


SI Figure S7: Contours of the tICA free energy surface of the CDR-H2 loop with the projected canonical cluster median crystal structures and the representative CDR-H2 loop ensemble in the background. On the right the color-coding of the canonical cluster medians is shown.


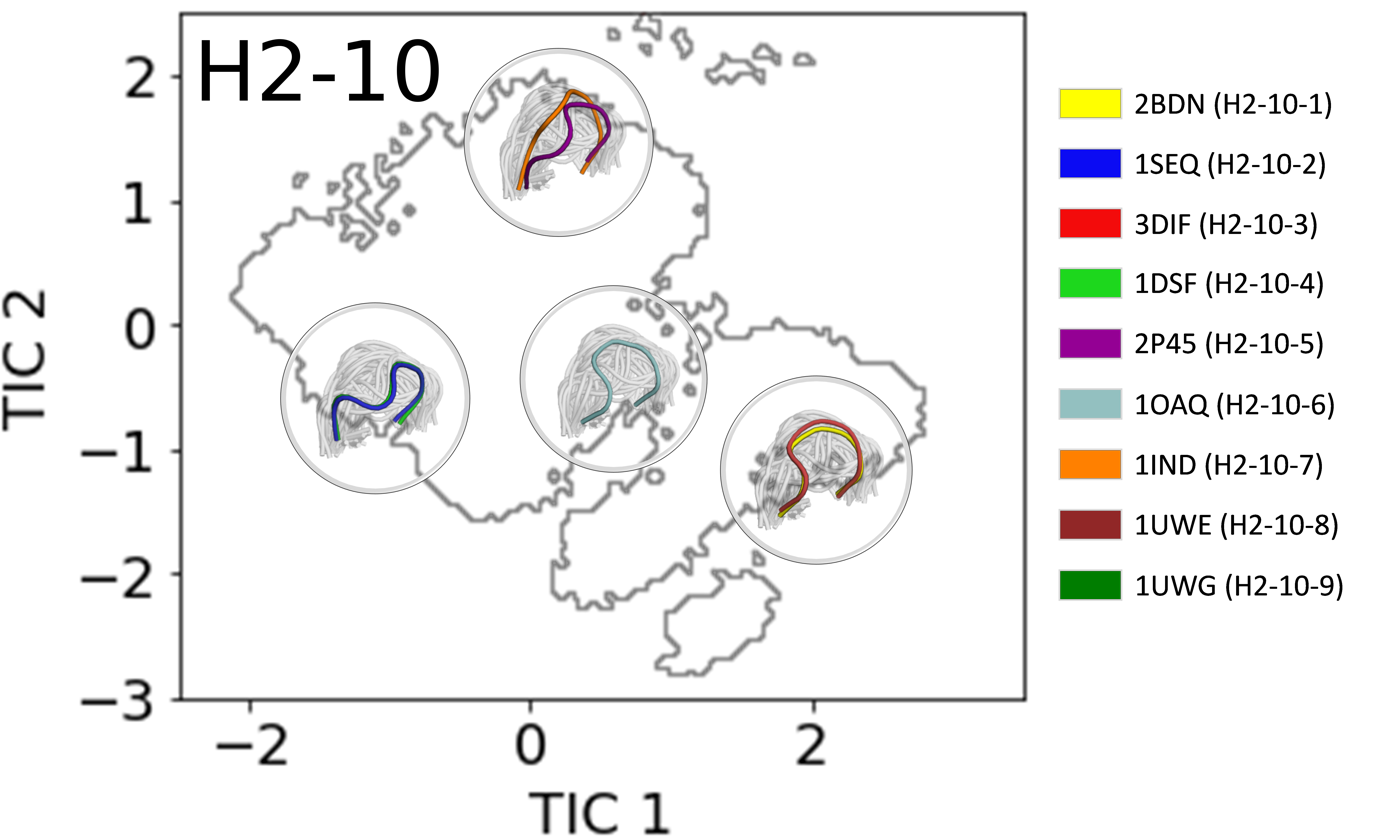


SI Figure S8: Contours of the tICA free energy surface of the CDR-H2 loop with the projected canonical cluster median crystal structures and the representative CDR-H2 loop ensemble in the background. On the right the color-coding of the canonical cluster medians is shown.


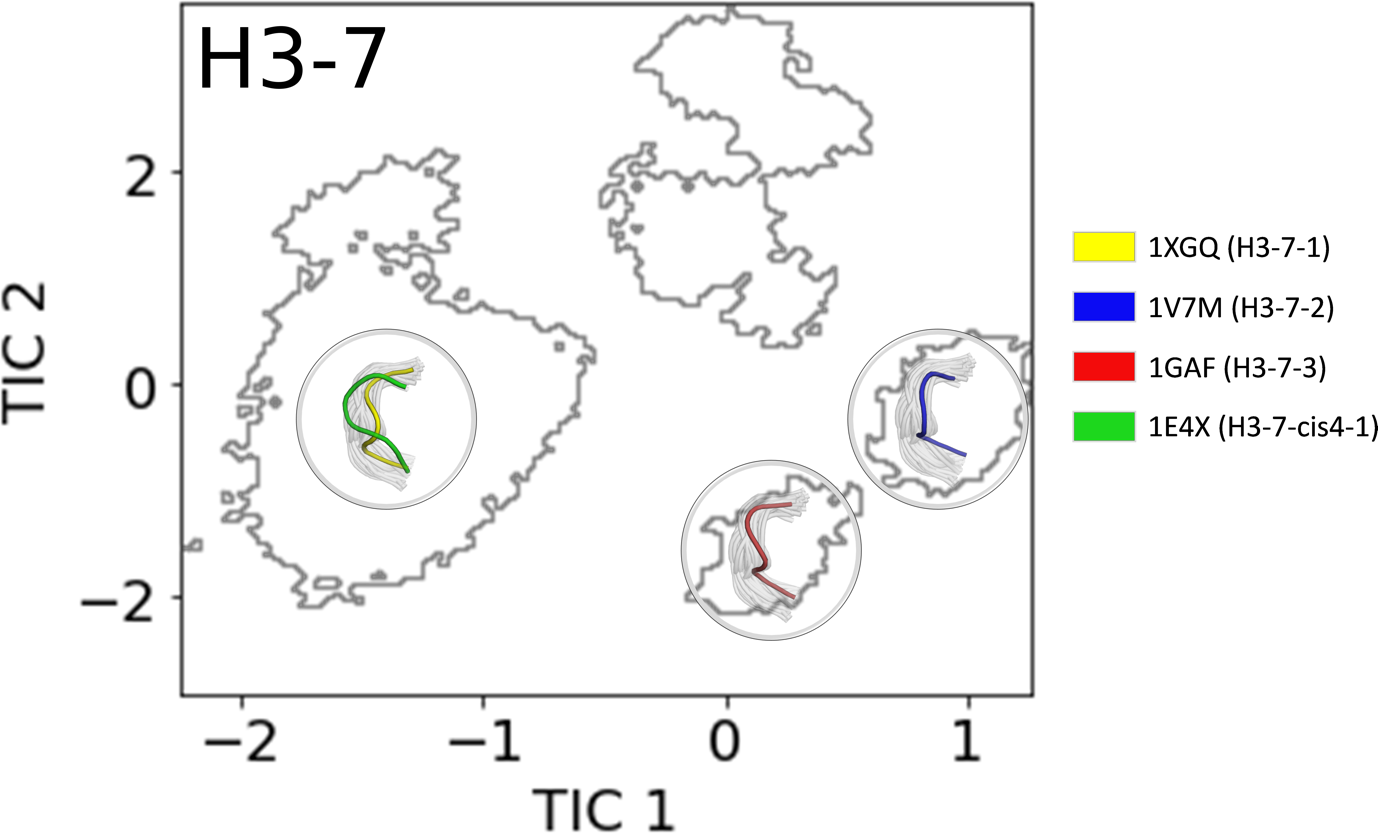


SI Figure S9: Contours of the tICA free energy surface of the CDR-H3 loop with the projected canonical cluster median crystal structures and the representative CDR-H3 loop ensemble in the background. On the right the color-coding of the canonical cluster medians is shown.


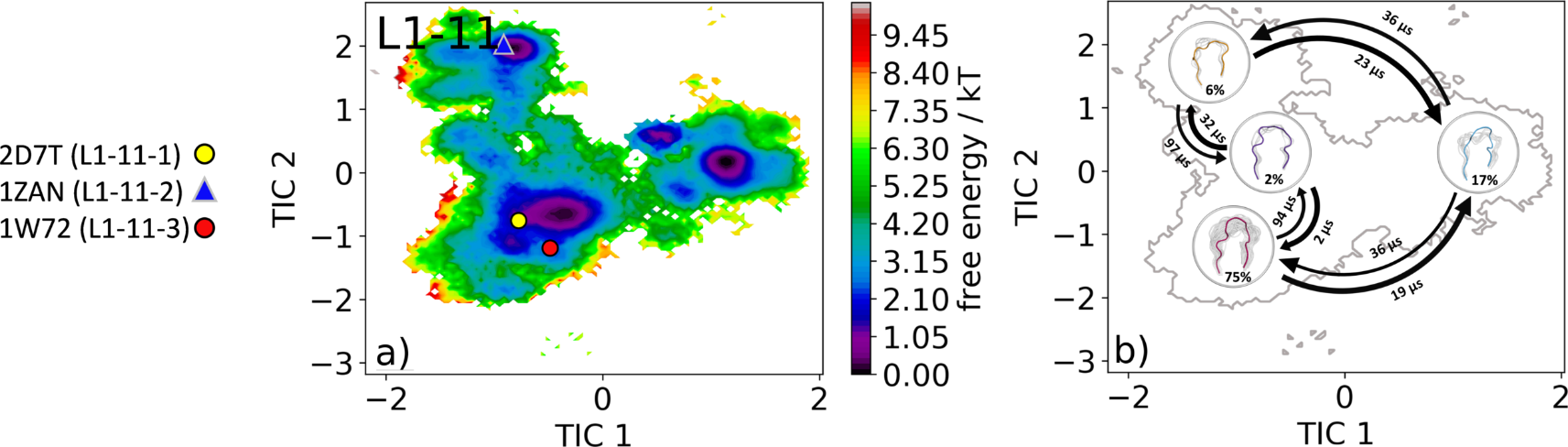


Figure 10: Free energy surface of the CDR-L1 loop with a loop length of eleven residues including the projected canonical cluster median representatives. The canonical cluster representative of the cluster L1-11-1 with the PDB accession code 1ZAN was used starting structure for the resulting 24.4 µs of trajectories. The canonical cluster representative used as starting structure for simulations is shaped as triangle, while all the other available canonical cluster median X-ray structures are visualized as circles and the respective color-coding is shown on the left. b) Contours of the free energy surface are displayed in the background of the Markov-state model. The macrostate representatives with the respective macrostate ensemble and transition kinetics are also included. The macrostate representatives were colored independent of the canonical cluster representatives in a) and summarize the kinetically relevant conformations of the CDR-L1 loop in solution. We obtained four macrostates, in which all canonical cluster medians are present.


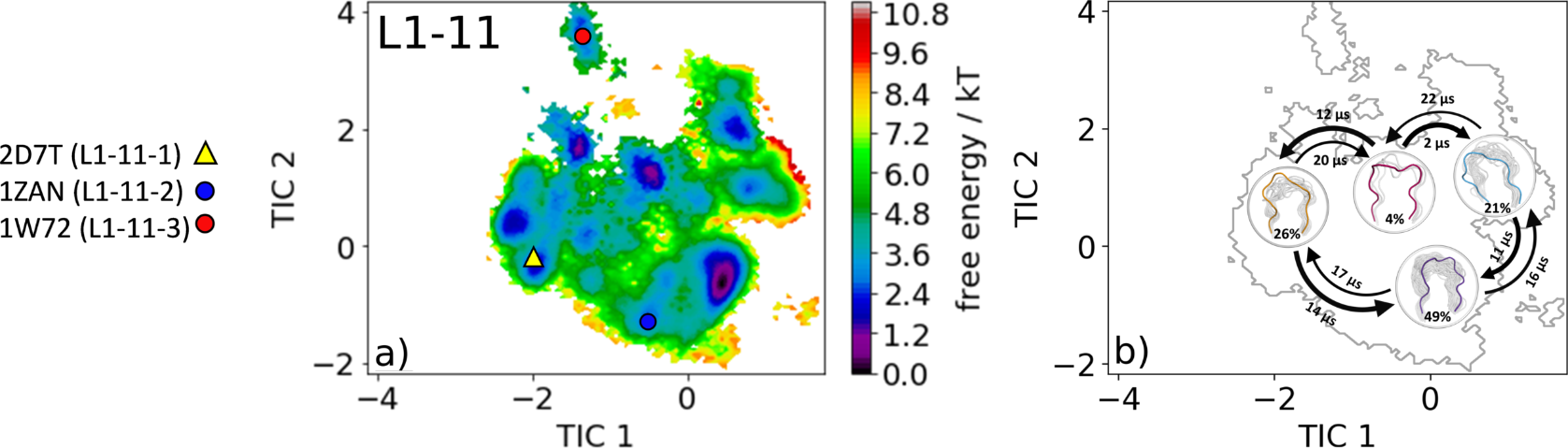


Figure 11: Free energy surface of the CDR-L1 loop with a loop length of eleven residues including the projected canonical cluster median representatives. The canonical cluster representative of the cluster L1-11-2 with the PDB accession code 2D7T was used starting structure for the resulting 17.4 µs of trajectories. The canonical cluster representative used as starting structure for simulations is shaped as triangle, while all the other available canonical cluster median X-ray structures are visualized as circles and the respective color-coding is shown on the left. b) Contours of the free energy surface are displayed in the background of the Markov-state model. The macrostate representatives with the respective macrostate ensemble and transition kinetics are also included. The macrostate representatives were colored independent of the canonical cluster representatives in a) and summarize the kinetically relevant conformations of the CDR-L1 loop in solution. We obtained four macrostates, in which all canonical cluster medians are present.


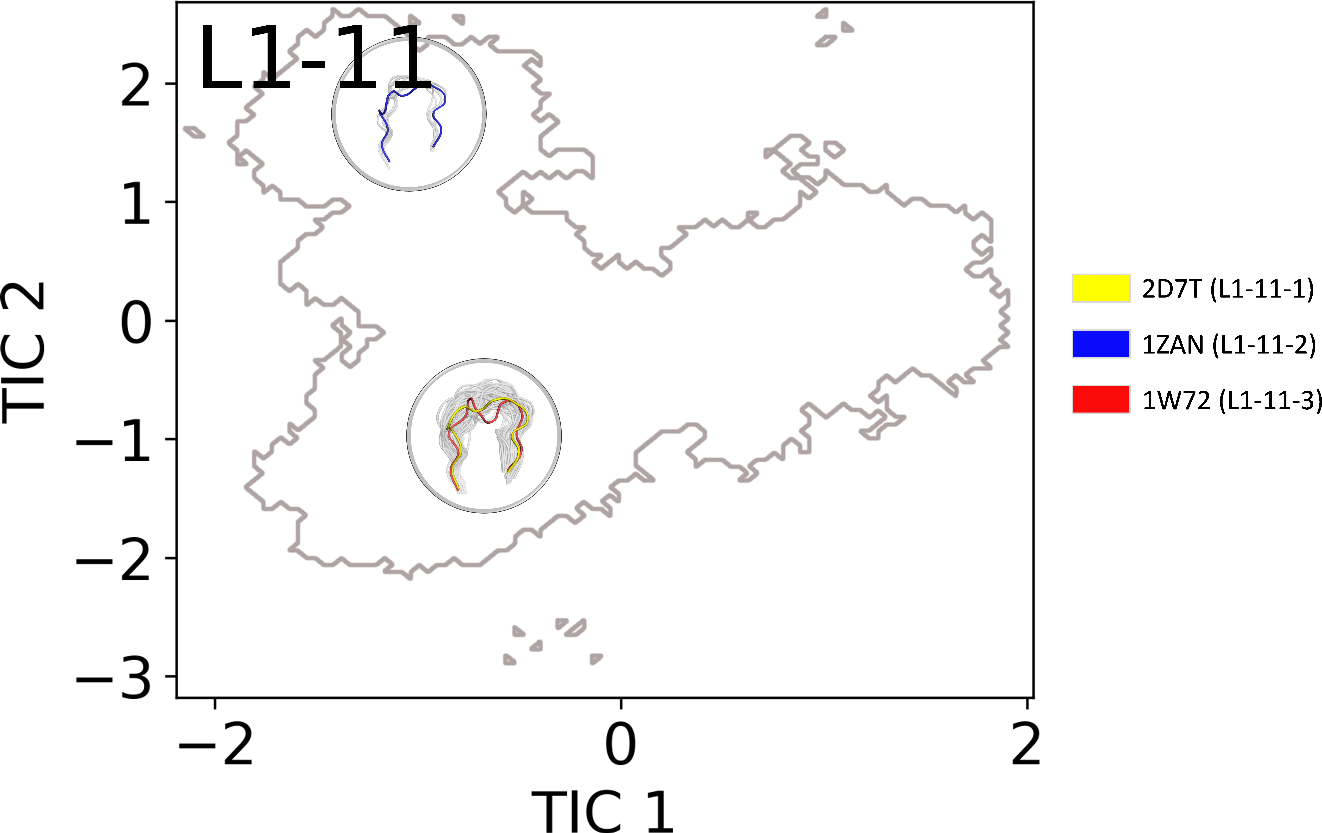


Figure 12: Contours of the tICA free energy surface of the CDR-L1 loop, using the canonical cluster representative of the cluster L1-11-1 (PDB accession code 1ZAN) as starting structure for molecular dynamics simulations. On the right the color-coding of the canonical cluster representatives with loop length eleven are shown and correspond to the structures shown in the tICA plot with the representative CDR-L1 loop macrostate ensemble in the background.


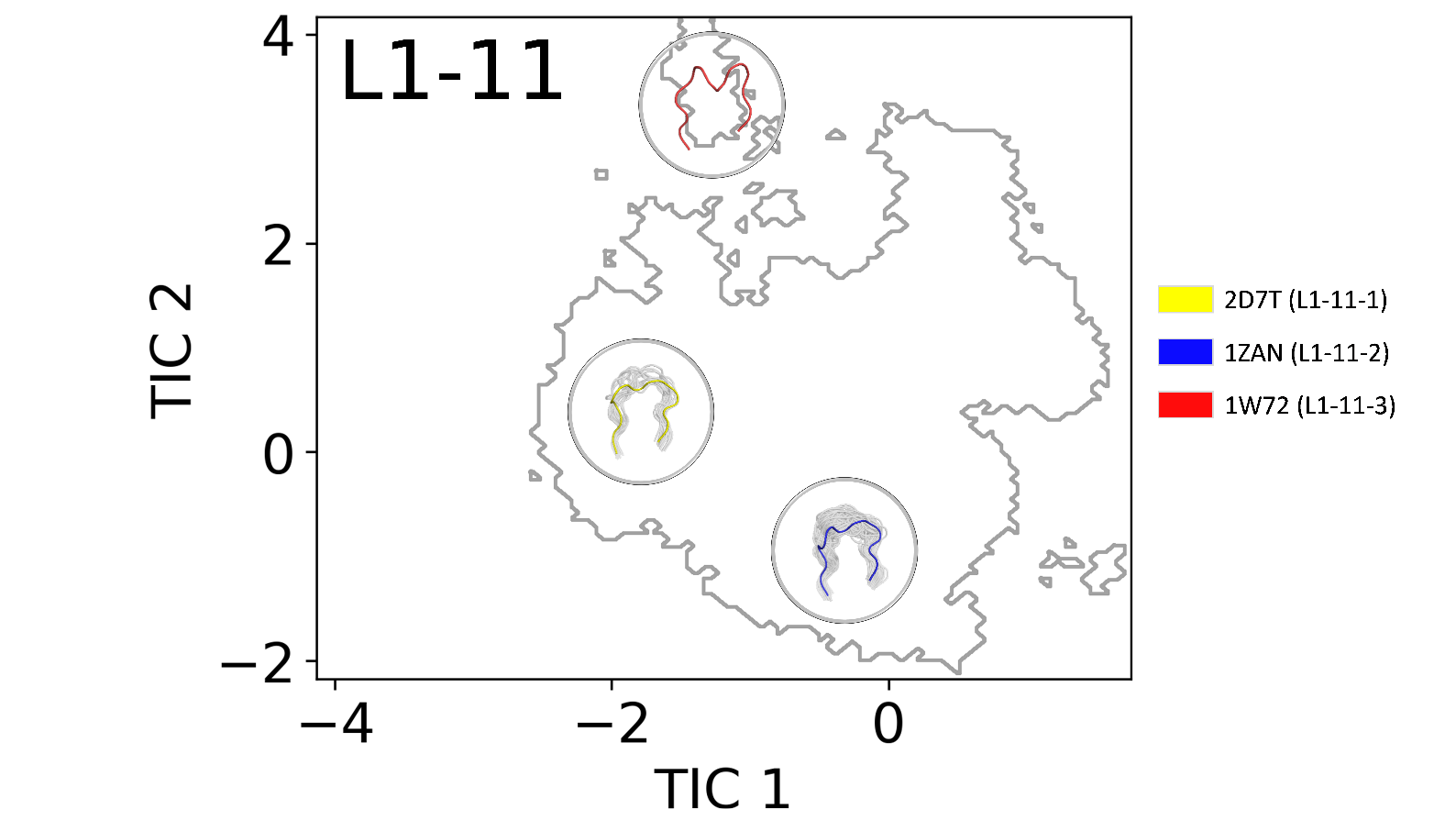


Figure 13: Contours of the tICA free energy surface of the CDR-L1 loop, using the canonical cluster representative of the cluster L1-11-2 (PDB accession code 2D7T) as starting structure for molecular dynamics simulations. On the right the color-coding of the canonical cluster representatives with loop length eleven are shown and correspond to the structures shown in the tICA plot with the representative CDR-L1 loop macrostate ensemble in the background.

*SI Table S1: RMSD values and dihedral angle distances for the canonical cluster centroids to their closest neighbour in our molecular dynamics simulations. In the heading the starting structure for the simulations are indicated.*

| **L1-11 (1W72)** | **Dihedral Distance / °** | **RMSD / Å** |
| --- | --- | --- |
| L1-11-3 (1W72) | 3 | 0.4 |
| L1-11-1 (1ZAN) | 27 | 1.1 |
| L1-11-2 (2D7T) | 25 | 1.1 |
|  |  |  |
| **L1-11 (1ZAN)** | **Dihedral Distance / °** | **RMSD / Å** |
| L1-11-3 (1W72) | 31 | 1.2 |
| L1-11-1 (1ZAN) | 6 | 0.2 |
| L1-11-2 (2D7T) | 21 | 0.3 |
|  |  |  |
| **L1-11 (2D7T)** | **Dihedral Distance / °** | **RMSD / Å** |
| L1-11-3 (1W72) | 27 | 1 |
| L1-11-1 (1ZAN) | 21 | 0.3 |
| L1-11-2 (2D7T) | 8 | 0.2 |
|  |  |  |
| **L2-8 (2FL5)** | **Dihedral Distance / °** | **RMSD / Å** |
| L2-8-4 (1ETZ) | 10 | 0.2 |
| L2-8-3 (1I8K) | 46 | 0.5 |
| L2-8-1 (1YEJ) | 3 | 0.1 |
| L2-8-5 (2AEP) | 6 | 0.5 |
| L2-8-2 (2FL5) | 6 | 0.2 |
|  |  |  |
| **L2-12 (3BJ9)** | **Dihedral Distance / °** | **RMSD / Å** |
| L2-12-2 (2OTU) | 16 | 0.7 |
| L2-12-1 (3BJ9) | 5 | 0.1 |
|  |  |  |
|  |  |  |
| **L3-9 (2FL5)** | **Dihedral Distance / °** | **RMSD / Å** |
| L3-9-1 (1F4X) | 35 | 1.1 |
| L3-9-cis7-2 (1G7I) | 23 | 0.4 |
| L3-9-cis7-1 (1J1P) | 4 | 0.1 |
| L3-9-2 (1KCS) | 15 | 0.4 |
| L3-9-cis7-3 (1L7I) | 15 | 0.5 |
|  |  |  |
|  |  |  |
| **H1-13 (1IC4)** | **Dihedral Distance / °** | **RMSD / Å** |
| H1-13-7 (1DQD) | 37 | 0.5 |
| H1-13-8 (1HCV) | 51 | 1.0 |
| H1-13-4 (1IC4) | 11 | 0.2 |
| H1-13-cis9-1 (1JTP) | 38 | 0.8 |
| H1-13-9 (1KXV) | 38 | 0.7 |
| H1-13-5 (1MVF) | 23 | 1.0 |
| H1-13-10 (1RHH) | 37 | 0.4 |
| H1-13-1 (1RUR) | 35 | 0.8 |
| H1-13-3 (1U0Q) | 34 | 0.6 |
| H1-13-11 (1UM5) | 40 | 0.6 |
| H1-13-2 (1UWG) | 43 | 0.7 |
| H1-13-6 (2P45) | 32 | 0.9 |
|  |  |  |
| **H1-13 (1HCV)** | **Dihedral Distance / °** | **RMSD / Å** |
| H1-13-7 (1DQD) | 53 | 1.2 |
| H1-13-8 (1HCV) | 31 | 0.5 |
| H1-13-4 (1IC4) | 20 | 1.0 |
| H1-13-cis9-1 (1JTP) | 29 | 1.2 |
| H1-13-9 (1KXV) | 29 | 0.7 |
| H1-13-5 (1MVF) | 39 | 1.1 |
| H1-13-10 (1RHH) | 56 | 1.3 |
| H1-13-1 (1RUR) | 38 | 1.0 |
| H1-13-3 (1U0Q) | 33 | 1.1 |
| H1-13-11 (1UM5) | 34 | 1.1 |
| H1-13-2 (1UWG) | 24 | 1.1 |
| H1-13-6 (2P45) | 36 | 1.4 |
|  |  |  |
|  |  |  |
| **H2-10 (2BDN)** | **Dihedral Distance / °** | **RMSD / Å** |
| H2-10-4 (1DSF) | 37 | 0.4 |
| H2-10-7 (1IND) | 13 | 0.4 |
| H2-10-6 (1OAQ) | 19 | 0.3 |
| H2-10-2 (1SEQ) | 23 | 0.2 |
| H2-10-8 (1UWE) | 23 | 0.3 |
| H2-10-9 (1UWG) | 47 | 0.6 |
| H2-10-1 (2BDN) | 6 | 0.1 |
| H2-10-5 (2P45) | 9 | 0.2 |
| H2-10-3 (3DIF) | 20 | 0.3 |
|  |  |  |
|  |  |  |
| **H2-10 (1DSF)** | **Dihedral Distance / °** | **RMSD / Å** |
| H2-10-4 (1DSF) | 3 | 0.1 |
| H2-10-7 (1IND) | 12 | 0.4 |
| H2-10-6 (1OAQ) | 8 | 0.2 |
| H2-10-2 (1SEQ) | 19 | 0.1 |
| H2-10-8 (1UWE) | 19 | 0.6 |
| H2-10-9 (1UWG) | 20 | 0.6 |
| H2-10-1 (2BDN) | 10 | 0.3 |
| H2-10-5 (2P45) | 5 | 0.1 |
| H2-10-3 (3DIF) | 17 | 0.4 |
|  |  |  |
|  |  |  |
| **H3-7 (1GAF)** | **Dihedral Distance / °** | **RMSD / Å** |
| H3-7-3 (1GAF) | 2 | 0.1 |
| H3-7-2 (1V7M) | 3 | 0.1 |
| H3-7-1 (1XGQ) | 7 | 0.1 |
| H3-7cis4-1 (1E4X) | 17 | 0.6 |

**Addition to the methods section:**

For the metadynamics simulations we used the version of gromacs-2019.2 together with the plumed-2.5.2. For the analyses we used cpptraj, the hierarchical clustering average linkage algorithm of the AMBER16 tools and clustered on the respective CDR loop residues by using a distance cut-off criterion of 1.2 Å. We used this resulting clusters (~150 clusters) as starting structures for each 100 ns of molecular dynamics simulations in an NpT ensemble. For the Markov-state model we used pyEMMA (version 2.5.6) and followed the guidelines of the pyEMMA tutorial. To construct the tICA we used as input features the backbone torsions of the respective CDR loop. These links to the tutorial and commands used to construct the MSMs (<http://www.emma-project.org/v2.4/generated/pentapeptide_msm.html> ; <http://www.emma-project.org/v2.4/generated/MSM_BPTI.html>). We also provided our macrostate representatives with decreasing state probabilities as PDB for each studied antibody fragment.
